# State budget transfers to health insurance funds: extending universal health coverage in low- and middle-income countries of the WHO European Region

**DOI:** 10.1186/s12939-016-0321-0

**Published:** 2016-04-02

**Authors:** Inke Mathauer, Mareike Theisling, Benoit Mathivet, Ileana Vilcu

**Affiliations:** Department of Health Systems Governance and Financing, World Health Organization, Avenue Appia, 1211 Geneva, Switzerland; Health, Population Policy, Social Security Division, Federal Ministry for Economic Cooperation and Development, Bonn, Germany; Consultant with the World Health Organization at the time of writing from October 2014 to December 2015, Avenue Appia, 1211 Geneva, Switzerland

**Keywords:** Universal health coverage, Health insurance, Informal sector, Vulnerable populations, Government budget transfers, Financial protection

## Abstract

**Background:**

Many low-and middle-income countries (LMIC) of the World Health Organization (WHO) European Region have introduced social health insurance payroll taxes after the political transition in the late 1980s, combined with budget transfers to allow for exempting specific population groups from paying contributions, such as those outside formal sector work and in particular vulnerable groups. This paper assesses the institutional design aspects of such financing arrangements and their performance with respect to universal health coverage progress in LMIC of the European region.

**Methods:**

The study is based on a literature review and review of secondary databases for the performance assessment.

**Results:**

Such financing arrangements currently exist in 13 LMIC of that region, with strong commonalities in institutional design: This includes a wide range of different eligible population groups, mostly mandatory membership, integrated pools for both the exempted and contributors, and relatively comprehensive benefit packages. Performance is more varied. Enrolment rates range from about 65 % to above 95 %, and access to care and financial protection has improved in several countries. Yet, inequities between income quintiles persist.

**Conclusions:**

Budget transfers to health insurance arrangements have helped to deepen UHC or maintain achievements with respect to UHC in these European LMICs by covering those outside formal sector work, and in particular vulnerable population groups. However, challenges remain: a comprehensive benefit package on paper is not enough as long as supply side constraints and quality gaps as well as informal payments prevail. A key policy question is how to reach those so far uncovered.

**Electronic supplementary material:**

The online version of this article (doi:10.1186/s12939-016-0321-0) contains supplementary material, which is available to authorized users.

## Background

Many low-and middle-income countries (LMIC) of the World Health Organization (WHO) European Region have introduced payroll taxes and a social health insurance (SHI) agency after the political transition in the late 1980s. Prior to this transition, these countries were characterized by high levels of financial protection and equity in access, as well as a broad population coverage. Thus, there was a risk of moving away from universal population coverage if they had merely shifted to or continued with “traditional” SHI that would focus on the formal sector employees and their dependents only, whereby entitlement is linked to paying contributions. Instead, government revenues were the starting point for health financing, and budget transfers were built into the design of the introduction of earmarked payroll taxes right from the start. Budget transfers primarily serve, though not exclusively, to exempt specific population groups from payroll taxes as they are unable to pay insurance contributions by themselves due to no, very low or unsteady income.

This option of using state budget revenues to explicitly pay the contributions on behalf of specific groups that do not contribute themselves, in addition to cross-subsidization from contributions, is also known as government subsidization of insurance contributions or as subsidized enrolment in health insurance in the literature, particularly in other regions [1–6]. More than 40 LMIC have chosen to progress towards universal health coverage (UHC) through this health financing arrangement. This is because they realise that progress towards UHC is not possible only via earmarked payroll taxes collected from the formal sector. Delinking entitlement from directly paying contributions and using general tax funding to maintain or expand coverage via a health insurance type mechanism, i.e. via a separate pooling and purchasing agency, is particularly widespread in the WHO European Region. While taking on this specific focus on state budget transfers to health insurance type schemes, this is not to say that this is the only viable health financing mechanism and path to progress towards and deepen UHC.

This paper specifically analyses these government revenue transfer arrangements with a regional focus on the low-and middle-income countries of the WHO European Region (abbreviated as EURO) (see [7] for a list of all countries in that region). Existing literature on this topic and region includes single country studies on the health (financing) system. Taking on a functional perspective, Kutzin et al. [8, 9] provide a comprehensive and detailed assessment of health financing reform implementation in transition countries of that region. Building upon this work, this paper focuses on the specific institutional design features of the contribution exemption arrangements using budget transfers in EURO LMIC.

The overall aim of this paper is to provide a focused and detailed overview of such arrangements and their institutional design aspects and to explore their patterns, commonalities and differences among these countries. It also seeks to assess progress with respect to UHC on the basis of which those critical institutional design features that are particularly conducive for UHC progress are identified. This serves to derive policy lessons of what works and what does not. Furthermore, this paper complements another paper by Vilcu/Mathauer (2016) [10] of the same subject on high-income countries of the WHO European Region. They are part of a series of regional studies (Europe, Asia, Latin America, Africa), all with the same research focus on budget transfers/subsidization arrangements [11, 12]. Last not least, even though the term "scheme" or "financing arrangement" is used, the unit of analysis is the whole system, since these schemes aim to cover and are designed for the whole of the population.

The focus of this paper is largely on population groups outside formal sector employment. These include poor people, agricultural and informal sector workers, as well as other vulnerable groups. In general, vulnerable groups can be defined as those individuals with an increased susceptibility to not having adequate access to health care and sufficient financial protection due to economic, demographic, or geographic characteristics [13].

The paper is structured as follows. The next section outlines the methods and the analytical framework applied. The third section assesses the institutional design aspects of these financing arrangements as well as progress towards UHC. This is followed by a discussion of possible effects of specific institutional design aspects on and challenges related to progress towards UHC, while the last section concludes with policy lessons.

## Methods

This paper included those European LMIC having a (social) health insurance type arrangement that operates as a separate purchasing agency with budget transfers serving to maintain and deepen coverage of selected population groups, in particular people outside formal sector work and vulnerable groups. In an initial step, all EURO countries belonging to the group of low- and middle income, based on the World Bank country classification of 2014 [14] were screened and included if the Global Health Expenditure Database (GHED) [15] reported Social Health Insurance expenditure (termed social security funds in GHED). In a second step, the literature was screened via PubMed and Google to identify all LMIC countries of that region with both social health insurance expenditure and government revenue transfers to exempt specific population groups. For that matter, the name of each country was combined with the following search terms: budget transfer OR health subsidization OR health subsidy OR health insurance OR health vulnerable. This two-step exploration rendered a list of 13 countries. While Latvia does not belong to this list, notably, since 2013 it prepares the introduction of a Compulsory Health Insurance system by linking entitlement to health services to the payment of income tax [14]. Kazakhstan is not included here, since the Mandatory Health Insurance Fund operated for only two years [16].

Once the list of countries was established, in a next step, a literature search was conducted for each country over the period of 1980 to May 2015 in PubMed, Science Direct, JSTOR and Google. Titles identified through the search process were reviewed, and if found to be relevant the abstract or executive summary was read. If this suggested that the publication could provide information on the institutional design or on its impact, the full publication was assessed. In Google, the first five pages, with 10 results per page, were considered. The search generated several publications for each country. While this allowed for triangulation, the study presents information from the most recent publications.

The same search terms were used in this step: health system OR health financing OR health insurance OR health subsidy OR budget transfer OR exemption OR health vulnerable OR health Roma. All key words were used in combination with the respective country AND/OR scheme name. The data search for information on UHC related performance was based on the following terms: impact health insurance OR catastrophic health expenditure OR impoverish* health care OR out-of-pocket payment OR financial protection OR access health OR utilization health care OR health insurance coverage OR universal coverage. Additionally, data for the performance assessment was collected from the WHO databases “Health for All” and GHED, country household income and expenditure survey reports, insurance statistics as well as other country statistics and reports. Thus, the study is based on a literature review and review of secondary databases for the assessment of UHC progress.

The paper’s analytical framework to assess the institutional design features of budget transfer arrangements starts from the three health financing functions described in Kutzin (2001) [17] and looks specifically at the following features:

1. Revenue raising:
  Eligibility and enrolment arrangements;
  Financing arrangements;
2. Pooling arrangements;
3. Purchasing arrangements and benefit package design.

On this basis, a more detailed framework was developed which is presented in Table 1 providing an overview of the institutional design aspects and how these potentially relate to progress toward UHC. These aspects and UHC progress indicators are explained in more detail in the Additional file 1. It is important to note that enrolment and coverage through exemption from contributions in such schemes is only one possible and plausible factor among several to explain improvements in the level of financial protection and access to care of subsidized beneficiaries.

**Table 1:** Institutional design features of state budget transfer/government subsidization arrangements

Institutional design aspect	Related policy choices	Intermediate output indicators	UHC related progress indicators
Eligibility and enrolment rules			
Groups eligible for exemption from contributions/subsidization	Definition of vulnerability (e.g. children, unemployed, pregnant women, informal sector workers, poor, near poor)	Share of the eligible among the bottom two income quintiles and other vulnerable groups	Total population coverage (i.e. enrolment in health insurance fund), differentiated along income quintiles
Targeting method	E.g. universal (based on a very broad criterion such as residence or no employment in the formal sector), indirect (based on socio-demographic, socio-economic or geographic characteristics usually correlated with poverty and vulnerability), direct (through a means assessment or proxy means testing); different targeting approaches can be in place at the same time for different groups	Share of the exempted/subsidized within total (insured) population; Share of the exempted/subsidized among those being targeted for exemption/subsidization (targeting effectiveness of the system)	
Enrolment process	Active enrolment by the beneficiary or automatic enrolment by the authorities		
Organization responsible for identification of the exempted non-contributors/the subsidized	E.g., insurance company; central, regional, local government		
Type of enrolment / membership	Mandatory or voluntary		
Financing arrangements			
Degree of subsidization/co-contribution	Full or partial (a co-contribution is required)	Share of the exempted/subsidized within total (insured) population/those being targeted for subsidization (importance of government revenue)	
Type of transfer mechanism	Individual-based (a specific amount is being paid for each exempted individual), or lump-sum (a lump sum transfer for the entire exempted population is made)		
Calculation logic to determine the amount of funds to be transferred	E.g., based on regular contribution levels, minimum or average wages, specific percentage of the government budget, negotiated by the government	Sufficient funding for a comprehensive benefit package Level of cross-subsidization from contributions	Financial protection (incidence of catastrophic^a^ / impoverishing health expenditure), also differentiated along income quintiles and other aspects; Access to services
Source of funding for state budget transfers	E.g. general government revenues, earmarked government revenues, transfers from other health insurance funds or from contributors within the same pool (cross-subsidization), donor funding		
Pooling arrangements			
Type of pool(s) (general)	Single pool, or multiple pools	Degree of fragmentation, Size and composition of pools, Level of cross-subsidization	Equity in access; Equity in financing; Financial protection
Type of pool (exempted/subsidized)	Exempted/subsidized integrated in the pool with contributors, or separate pool for the exempted/subsidized		
Type of health insurance affiliation/ membership of the contributors	Voluntary or mandatory		
Purchasing arrangements and benefit package design			
Range of services covered by the benefit package	E.g. comprehensive, inpatient focus, outpatient focus, pharmaceuticals, dental care, indirect costs (e.g. transportation) Different or same package as that for contributors		Financial protection; Access (utilization rates); Equity in access
Degree of cost-sharing	Cost-sharing mechanisms (e.g., co-insurance, co-payment, deductible) and rates		
Provider payment mechanisms	Type of provider payment and rates Same or different rules around provider payment	Efficiency	

^a^ As per the WHO definition, catastrophic expenditure “occurs when a household’s total out-of-pocket health payments equal or exceed 40 % of household’s capacity to pay” ([59], p. 4)

Source:[11]

## Results

### Country and scheme overview

This paper includes 13 countries in the European region with a health insurance type scheme plus government revenue transfers, i.e. Albania, Bulgaria, Bosnia & Herzegovina, Georgia, Kyrgyzstan, Lithuania, Montenegro, Republic of Moldova, Romania, the Russian Federation, Serbia, The Former Yugoslav Republic of Macedonia (TFYR Macedonia) and Turkey. This is a mix of both lower- and upper-middle income countries, with Kyrgyzstan having moved to the group of lower middle-income countries only in 2013. The Russian Federation and Lithuania, high-income countries since 2012 only, are also included as a way to capture their reform experiences over the past 15 years since introduction.

Bosnia & Herzegovina consists of two separate entities: the Federation of Bosnia & Herzegovina and the separate Republika Srpska, with both entities having their own separate health insurance pool. Therefore, this study covers in total 14 country health financing systems in 13 countries.

With the exception of Turkey, the countries assessed here have a similar legacy with respect to financing their health systems falling into two patterns. In Georgia, Kyrgyzstan, Lithuania, the Republic of Moldova and the Russian Federation, as part of the former Soviet Union, health care was tax-funded from the government budget. The same approach was taken by Albania, Bulgaria and Romania [6]. All these countries, except the Republic of Moldova, introduced payroll taxes and combined these with government revenue transfers in the early or mid-1990s as part of other major economic reforms. The Republic of Moldova introduced both payroll taxes and government revenue transfers later, in 2004. The introduction of earmarked payroll taxes foremost served to generate sufficient public revenues to increase coverage as to scope (i.e. the range of services) and depth (proportion of costs covered). On the other hand, in Bosnia & Herzegovina, Montenegro, Serbia, and TFYR Macedonia, which had belonged to the Socialist Republic of Yugoslavia, state employees were covered by compulsory health insurance prior to the transition [18]. Thereafter, SHI coverage was expanded to the whole population through the combination with state budget transfers. Before the collapse of the Eastern Bloc, these countries were characterized by broad population coverage, high levels of financial protection and equity despite problems like lack of financial resources, inefficiencies in resource allocation and material shortages [19]. This favourable “starting point” in terms of equity is a major difference to LMIC in other regions [20].

In contrast, Turkey and Georgia pursued a different path in that their financing arrangements for the exempted groups were implemented independently from the financial protection mechanisms for the rest of the population. In Turkey, social security existed for formal sector employees since the 1940s, whereas the “Green Card Scheme” for uninsured low income people was introduced in 1992 only. In 2012, this scheme was merged with the formal sector SHI schemes. As to Georgia, the government had created the “Medical Insurance Scheme for the Poor” (MIP) in 2006 [21], however until early 2013 the large majority of the population either had to pay for health care services out-of-pocket or could purchase voluntary private insurance. In September 2012, a comprehensive MIP-style benefit package for pensioners, children under 5 years and students was introduced, and in February 2013, the Georgian government set up a Universal Health Insurance Program. This Program targets every citizen that did not yet have insurance coverage up to that date, including vulnerable groups previously not eligible in the MIP [22]. Current plans focus on integrating the total population into one fund. Evaluations and analyses on these very recent developments in Georgia and Turkey are scarce, such that this paper primarily focuses on discussing the MIP and the Green Card Scheme.

### Eligibility, targeting and enrolment

Whereas the terms “health insurance subsidization” and “the subsidized” is appropriate for other regions, the developments in EURO are better grasped by a different terminology. Except for the former Yugoslavian countries, the majority of the population was covered prior to the transition. Hence, the shift to payroll taxation of formal sector employees and a contributory SHI system implied that various population groups outside the formal sector who did not or could not contribute, needed to be exempted from contributions in order to remain entitled. To capture this context, this paper rather talks of the “exempt” (exempted from contributions) instead of “the subsidized” (their contributions being subsidized). Exemption based on state budget transfers needs to be distinguished from family insurance. This refers to non-contributory coverage of family dependents, i.e. children and non-working spouse, via the principal affiliate through cross-subsidization among contributors, which is also in place in a number of countries of this region.

Regarding eligibility and related enrolment rules, strong commonalities prevail, with a few exceptions: In eleven out of thirteen countries, membership is de jure mandatory for the exempt. Only in the case of Georgia’s MIP and Turkey is participation voluntary [21, 23].

Second, some form of targeting is applied in nearly all countries in order to identify individuals eligible for exemption. All countries, except the Russian Federation, Turkey and Georgia employ indirect targeting based on demographic and socio-economic characteristics (see Table 2). The six population groups most frequently exempted include the unemployed (ten countries), disabled people (nine countries), recipients of social assistance (nine countries), youths enrolled in secondary or higher education (seven countries), pregnant women/postpartum mothers (seven countries) and children below a set age ranging from 15 to 18 years (eight countries) or, as in the case of Montenegro, children whose parents are not able to work. Some of these population categories need to be registered prior to being able to enrol, e.g. the unemployed, disabled, pensioners and recipients of social assistance.

**Table 2:** Eligible groups for exemption from health insurance contributions

Country (World Bank income classification, 2014)	Year of introduction of (social) health insurance [7]	Year of introduction of government revenue transfers [7]	Eligible groups	Targeting method
Albania (UM)	1995	1995	children; women working at home; pregnant women; disabled people; cancer patients; unemployed; recipients of social assistance; elderly; war veterans [44, 60, 61]	indirect targeting [60]
Bosnia & Herzegovina (UM) - *Federation*	1997	1997	disabled people; unemployed; refugees [61]	indirect targeting [61]
Bosnia & Herzegovina (UM) – *Republika Srpska*	1999	1999	children < 15 years; mature students while registered with the Republic Bureau of Employment; pregnant women; disabled people; registered unemployed with secondary and higher education; redundant employees still receiving compensation in accordance with the labour; recipients of social assistance; refugees and displaced persons; elderly > 65 years [34, 61]	indirect targeting [61]
Bulgaria (UM)	1998	1998	children < 18 years; youths < 26 years enrolled in full-time education; pregnant women; postpartum mothers; disabled people entitled to social support; parents or spouses taking care of disabled people in constant need of help; unemployed entitled to compensation; refugees; prisoners; spouses of soldiers participating in international missions; injured while performing their duties as employees of the Ministry of Interior and civil servants; war veterans [38]	indirect targeting [38]
Georgia *(MIP)* (LM)	1995 (but abolished in 2004)	2006 *(Medical Insurance for the Poor)*	poorest 20 % of Georgian households two regions (Adjara and Tbilisi) fund additional beneficiaries (the near-poor, or those with slightly higher proxy means test scores) [21]	direct targeting: proxy-means test [21]
Kyrgyzstan (LM)	1997	1997	eligible since 1997: registered unemployed; people with disabilities since childhood; persons receiving social benefits eligible since 2000: children < 16 years; enrolled school children < 18 years; enrolled students of basic, secondary, and higher full-time education < 21 years eligible since 2002: refugees [30, 62, 63]	indirect targeting [30]
Lithuania (H)	1997	1997	children < 18 years; students; women on maternity leave; disabled and their carers; persons with certain illnesses; people on long-term sickness benefits; registered unemployed; recipients of pensions, recipients of social assistance and social insurance cash benefits [64, 65]	indirect targeting [66]
Montenegro (UM)	1993	1993	children of parents not able to work; orphans; unemployed entitled to unemployment benefits; recipients of social assistance; refugees; prisoners; military invalids; civil invalids of war; persons receiving veteran allowance if not otherwise insured [67, 68]	indirect targeting [68]
Republic of Moldova (LM)	2004	2004	children < 18 years; youths enrolled in full-time education; full-time students in mandatory postgraduate training and doctoral candidates; carers for severely disabled children (into adulthood); pregnant women; postpartum mothers; mothers with four or more children; registered disabled; registered unemployed (for a max. of six months); since 2009: recipients of social assistance according to the Law on Social Aid and families living below the poverty line [39] partial exemption for self-employed (50–75 %) [26, 49]	indirect targeting [26]
Romania (UM)	1999	1999	children < 18 years; youths < 26 years if students with no income; pregnant women; postpartum mothers; women on maternity leave; parents on leave for taking care of children < 2 years (or < 3 years if disabled); disabled; people on long-term sickness benefits; people with no income and having certain illnesses; unemployed; recipients of social assistance; prisoners; persons persecuted by the communist regime or declared war heroes in the 1989 Revolution; war veterans; retired persons with < 340 US$ income/month [24, 69]	indirect targeting [24]
Russian Federation (H)	1993	1993	non-working population with citizenship/ legal residence [27]	universal [27]
Serbia (UM)	1992	1992	children < 15 years; children/youth < 26 years if enrolled in education; pregnant women; postpartum mothers; disabled; registered unemployed; recipients of social assistance; beneficiaries of accommodation at institutions for social care; internally-displaced people; refugees; Roma population who due to the traditional way of life do not have a permanent living address; family members whose bread giver is engaged on regular military service; elderly > 65 years [70–72]	indirect targeting [72]
TFYR Macedonia (UM)	1991	1991	disabled; unemployed registered by the Employment Office; beneficiaries of basic social care; prisoners; war-disabled persons; [73]	indirect targeting [73]
Turkey *(Green Card Scheme)* (UM)	1950–1971	1992 *(Green Card Scheme)*	Turkish citizens living in Turkey who are not covered by any social security scheme and who have a per capita household income of < 1/3 of the minimum wage threshold (except for taxes and social security premiums); pensioners > 65 years and people with chronic illnesses are eligible even if their household’s per capita income is > 1/3 of the minimum wage [23]	direct targeting: proxy-means test [23]

^a^ At this time Montenegro was still part of the Federal Republic of Yugoslavia

*LM* Low middle income category, *UM* Upper middle income category, *L* Low income category, *H* High income category

The references used for each country are indicated in brackets

The Russian Federation does not use targeting. Eligibility has been “universalist” from the beginning, i.e. the entire population outside formal sector employment is exempt from paying contributions.

Only two countries (Georgia’s MIP and Turkey) apply direct targeting and use proxy-means testing, whereby individuals have to apply to be screened for eligibility. Yet, since September 2012, the Georgian government has been undertaking efforts to broaden coverage. First, it started to finance a benefit package comparable to that of the MIP to additional groups, namely pensioners, children under five years and students, by applying an indirect targeting approach [21]. In a second step, in 2013, the non-contributory Universal Health Care Program was introduced, pursuing a universalist approach. The Program intends to eventually cover the total population that is not otherwise insured via voluntary or corporate insurance [22].

To summarize, in the majority of the countries entitled individuals are identified by indirect targeting, based on a broad range of socio-demographic and socio-economic characteristics. Affiliation is most often mandatory. Turkey is different in this regard by employing direct targeting and making participation voluntary, which was equally the case for the previous MIP in Georgia.

### Financing arrangements

The SHI agencies have become the key pooling and purchasing agencies for most countries studied here: SHI expenditure is one form of general government revenues for health (GGHE), and the shares of SHI as of GGHE are above 70 % in nine countries in 2013. Three countries (Kyrgyzstan, Georgia, Turkey) have shares above 60 %, while the Russian Federation is lower with 39 % (see Table 3). Notably, the SHI expenditure, as reported in current GHED data, includes the general government transfers. However, the share of GGHE as of THE has only increased in 6 countries, while it actually decreased in 7 countries [15].

**Table 3:** Financing arrangements

Country	Type of transfer logic	Calculation logic to determine state budget transfer amounts	Financing source of government revenue transfers	Social Security Funds as % of GGHE (2013) [15]
Albania	lump-sum [31, 44]	base for calculating government transfer is per capita health care expenditure during the previous year [41]	central government budget [31]	74.1
Bosnia & Herzegovina	*Federation:* per capita (for unemployed) *Republika Srpska:* per capita [61]	*Federation:* n/a *Republika Srpska:* contributions from the entity budget may not be less than the average per person contribution from the previous year [61]	unemployed entitled to benefits: Unemployment Fund all other exempted: entity budget [61]	97.5
Bulgaria	per capita [38]	unemployed entitled to benefits: 8 % of the unemployment benefit (needs to be between the minimum and maximum income basis for insurance contributions), others: 8 % of a predefined minimum income basis for insurance contributions [38]	unemployed entitled to benefits: Unemployment Fund all other exempted: central government budget [38]	76.4
Georgia *(MIP)*	per capita [21]	contribution is determined through a tender process where insurance companies propose a contribution at which they would provide insurance covering a predefined benefit package; the insurance company with the lowest offer is selected [21]	central government budget two regions fund additional beneficiaries out of their regional government budgets [21]	68.8
Kyrgyzstan	per capita [63]	base for calculation is 1.5 times the minimum wage [63]	central government budget [63]; share of government transfers as of SHI revenues: 78 % (includes funding for the state guaranteed benefit package for all) [48]	64.1
Lithuania	per capita [75]	up to 36 % of the average gross monthly wage lagged by 2 years [76] Counter-cyclical measures of compulsory health insurance contributions made by the state on behalf of the unemployed and economically inactive people	central government budget [77]; share of government transfers as of SHI revenues: 48 % (calculated by the authors based on data from [76]	85.1
Montenegro	per capita [68]	unemployed entitled to benefits: 7.5 % of the unemployment benefit others: determined by law [61, 68]	unemployed entitled to benefits: unemployment fund: all other exempted: central government budget [61, 67]	89.3
Republic of Moldova	lump-sum [26]	at least 12.1 % of the total government budget (annually) [26]	central government budget [26] share of government transfers as of total SHI budget: 55 % [78]	85.0
Romania	women on maternity leave, people on sick leave, the unemployed, recipients of social assistance, prisoners: per capita [24] others: n/a	woman on maternity leave, women on leave to take care of children < 2 years (<3 years if disabled), prisoners: 6.5 % of the sum representing the value of two national minimum gross wages people on sick leave, unemployed, recipients of social assistance: 6.5 % of the sick leave, unemployment benefit or social assistance [24] others: n/a	women on maternity leave, prisoners, persons in military service: central government budget; people on sick leave: social security budget; unemployed: unemployment fund; recipients of social assistance: local government budget [24] all other exempted: n/a	83.0
Russian Federation	lump- sum [27]	per-capita amount of transfers is not specified; decided by the government (budget contributions not enough to cover the costs of the exempted population considering that utilization is also much higher compared to the contributors) [19]	regional government budgets [27]	38.9
Serbia	per capita [61]	15.9 % of the average wage (the government not always gives adequate funding for the exempted) [61]	disabled people: Pension and Disability Fund; all other exempted: central government budget [61, 72]	93.6
TFYR Macedonia	per capita [79]	unemployed entitled to benefits: 10 % of the benefit recipients of social assistance, prisoners, war invalids, families of fallen soldiers: 10 % of half the average wage all other exempted: 5.4 % of half the average wage [79]	the unemployed: unemployment fund all other exempted: central government budget [79]	91.6
Turkey *(Green Card Scheme)*	lump-sum [23]	n/a	central government budget [23]	64.1

Currently available health account data does not provide information on the shares of different sources of funds received by the pooling agency, but for the few countries where data was found the amount of government revenues being transferred is more than 50 %.

The main source for funding on behalf of the exempted groups are general government revenues, most often from the central government, with the exception of the Russian Federation and Bosnia & Herzegovina, which use regional government funds (see Table 3). Another important financing source are social security or social assistance funds, e.g. the unemployed fund for the unemployed or the disability fund for the disabled, whereas in Romania, funds to cover the eligible group of social assistance beneficiaries come from local government revenues [24].

The regional funding as a source for government revenue transfers together with its fragmented nature in funding can be considered as a main reason for the difficulties of the Russian SHI scheme to take-off, more so as it was launched in a period of deep economic crisis. The number of individuals meant to be exempted from payment of health insurance contributions greatly exceeded the funding capacity of the regional budgets, themselves experiencing a massive shrinking of their fiscal space [25]. This triggered either a lowering of regional/local budget funding to providers or arrears of payment to the mandatory health insurance system on behalf of the exempted population, ultimately leading to high enrolment rates, but with poor benefits. This is still observable today. The initial difficulties in SHI implementation contributed to its weakening, and the objective that general government health expenditure should predominantly be channeled through the SHI was not achieved to date.

Notably, in all countries, eligible population groups are fully exempted. Full exemption is also motivated by the concern that even small contributions might represent a financial burden for these selected groups preventing them from enrolling.

However, there is variation with respect to the type of budget transfers on behalf of the exempt (see Table 3). These are provided in form of a lump-sum in Albania, the Republic of Moldova, the Russian Federation and Turkey. In contrast, a per-capita transfer logic applies in all other countries (in Georgia's MIP until 2014), i.e. a flat amount on behalf of each exempted member is transferred. In Romania, transfers are also per capita, but different amounts are paid for different groups.

Different approaches and formulas to determine the lump sum or per capita transfers are used:

1. For those groups that also receive social security benefits (e.g., the unemployed, social assistance recipients, etc.), the social security institutions pay a percentage of the respective benefit as a “contribution” to the health insurance fund. This is the case in Bulgaria, TFYR Macedonia, Montenegro, Romania and the Federation of Bosnia & Herzegovina. Contribution rates are the same as those of contributing members and range from 1 to 10 % of social security/social assistance benefits. These are not state budget transfers in the narrow sense, but part of general government revenue.
2. The calculation of the government’s contribution for the exempted can be based on the minimum wage, as in Kyrgyzstan and Romania, on the average wage, such as in TFYR Macedonia and Serbia, or the average contribution amount of the previous year, such as in the Republic Srpska. Alternatively, an amount is determined in Bulgaria.
3. A specific share of the government budget is allocated to the insurance fund(s) on behalf of the exempted population, such as in the Republic of Moldova, where the target share of allocations from the annual government budget aims to be at least at 12 % [26].
4. The contribution level is not specified and is instead set on the basis of a negotiation process by the government. This is the case in Montenegro (for those groups not receiving social security benefits) and the Russian Federation. This procedure causes difficulties to cover the costs of the exempted population since the amount transferred to the health insurance fund is not necessarily linked to the actual expenditure [19, 27].
5. For Georgia’s MIP, the contribution was determined through a tender process. Private insurance companies proposed a contribution rate for which they were willing to offer a predefined benefit package for individuals eligible for the MIP. The insurance with the lowest offer was then selected to provide the MIP package to beneficiaries [21].
6. In Albania, the base for calculating the government transfer is the per capita health care expenditure during the previous year.

To summarize, eligible population groups are fully exempted from making contributions in all countries. The source of state budget transfers are primarily central government revenues or to a lesser extent social security funds. In most of the countries the transferred amount is based on a defined calculation formula and per capita.

### Pooling arrangements

One of the most notable commonality in institutional design is that all countries operate an integrated pool covering both the exempted and contributors right from the beginning when the health insurance agency was established, with Turkey and Georgia also having merged their schemes into a single pool in 2012 and 2013 respectively.

Bosnia & Herzegovina and the Russian Federation are the only two countries with multiple funds along territorial lines, whereby the exempted population is integrated into and part of the same territorial fund as the rest of the contributing members. A risk equalization mechanism is in place in the Russian Federation and since 2002 in the Federation of Bosnia & Herzegovina [28], with the aim of enhancing equity in the redistributive capacity across territorial funds. In Russia, however, in combination with a lack of clarity of the equalization rules, the available funding at the federal fund level proved to be insufficient to compensate for differences in regional revenues from contributions and regional budget transfers for the exempt [29]. Later on, federal and to a lesser extent regional budgets outside the health insurance system were used for the purpose of risk equalization [27].

To sum up, in all countries, the exempted are meanwhile part of the same fund as the formal sector employees, and government revenue transfers are pooled together with the contributions of formal sector employees. This takes place within a single payer system, except in two countries.

### Purchasing

#### Provider payment mechanisms

Since the exempted population groups are part of the same scheme as the contributing members, except for Georgia and Turkey in the past, health services provided to the exempted are being remunerated through the same provider payment mechanisms and rates as those for the contributors. Providers are thus not confronted with two different sets of incentives for those two groups. More so, in Kyrgyzstan, providers receive a higher remuneration rates for those beneficiaries exempted from co-payments [30]. In contrast, in Georgia prior to 2013, the private health insurance schemes had differentiated provider payment rates for the MIP beneficiaries compared to the other categories of insured.

#### Scope of services covered by the benefit package

Overall, the range of services covered by the benefit packages is quite comprehensive in these countries, including – at least de jure – primary care, specialised outpatient care and inpatient services (see Additional file 2). However, there is still variation between countries regarding the precise setup of the benefit packages. In all countries, except Albania, Kyrgyzstan and the Republic of Moldova, the insurance schemes purchase the bulk of health care services. Apart from primary care, specialised outpatient care and inpatient services, the packages also include at least a limited range of preventive and emergency dental services (except in Georgia). The schemes in the Federation of Bosnia & Herzegovina, TFYR Macedonia and Montenegro also reimburse travel expenses related to seeking health care. Moreover, pharmaceuticals from the country’s list of essential medicines or a positive drug list are covered. Medicines coverage is more limited in the Russian Federation, and outpatient drugs are only reimbursed for specific population groups [27]. Importantly, in Bosnia & Herzegovina, Bulgaria, Lithuania, TFYR Macedonia, Romania, the Russian Federation and Turkey, emergency care and some key services are provided by the Ministry of Health (e.g. treatment of communicable diseases with outbreak potential, family planning) and can be accessed by everyone, regardless of insurance status.

A different approach has been chosen by Kyrgyzstan, where the government finances a so-called state-guaranteed benefit package for the entire population, whereas contributions and state budget transfers for the exempt are used to finance additional services for those enrolled [30]. The health insurance fund also operates as the purchaser of the state-guaranteed package [9].

However, in contrast to the legal provisions, the actual benefit package is in many countries de facto often less comprehensive due to lack of financial resources, health care facilities, qualified health care personnel, as well as supply side shortages [32]. Comparing the benefit package of the contributors with that of the exempted reveals that the benefit packages are identical in seven countries (Bosnia & Herzegovina, Bulgaria, Kyrgyzstan, Lithuania, Montenegro, Romania, and Turkey (see Additional file 3). In five countries (Albania, TFYR Macedonia, Republic of Moldova, Serbia and the Russian Federation), specific groups of the exempted, namely children and pregnant women, are covered by an even more comprehensive package. For example, additional dental care or outpatient medicines are included. Notably, in none of the countries is the benefit package for exempt smaller than the benefit package for the contributors.

#### Cost-sharing mechanisms

Cost-sharing for health care services and pharmaceuticals is common across the region. Many countries of the former Soviet Union and former Yugoslavia introduced cost-sharing mechanisms during the transition period in the early 1990s in order to raise additional revenues for the health sector [33]. Today, the Russian Federation is the only country without any formal cost-sharing for the benefit package other than for outpatient medicines in this region [27]. Elsewhere, a range of different cost-sharing mechanisms are applied, such as user charges per service or co-insurance, as well as cost-sharing ceilings and benefit ceilings for defined services (see Additional file 3). Co-insurance rates, for example, range from 10–50 % depending on the service and the population group.

A comparison of cost-sharing rates between the contributors and the exempted reveals that these two groups fall in principle under the same cost-sharing rules (see Additional file 3). Yet, in the majority of the countries, namely Albania, the Federation of Bosnia & Herzegovina, Bulgaria, Kyrgyzstan, Lithuania, TFYR Macedonia, Republic of Moldova, Romania and Serbia, certain groups among the exempt benefit from full or partial cost-sharing exemptions. Groups exempted from cost-sharing include children, pregnant women, chronically sick or disabled people, the unemployed and social assistance beneficiaries. However, nowhere are all population groups exempted from contributions fully exempt from cost-sharing. Specifically, in the Federation of Bosnia & Herzegovina the degree of cost-sharing is adapted to the social status and the income situation of an individual [34]. The same holds for Kyrgyzstan where the amount of co-payments varies depending on the region, disease profile, social status and existence of a referral [30]. Turkey was the only one where exempted individuals had to make co-payments for certain services that are fully covered for regular contributing members, namely dental care, prosthetics, and orthotics [23].

The real question, though, is whether the cost-sharing exemption policies are put in practice. Evidence from Romania, for example, suggests that they are not effective in protecting the poor groups from financial hardship, as three out of four poor patients do pay for health care [35].

Apart from formal cost-sharing payments, informal payments in the health sector have been reported in all the countries assessed here. Since informal payments are unpredictable, they might especially represent a high burden for poorer patients. Informal payments do not necessarily take the form of cash, but may be provided as presents, food or medical supplies taken to the health care facility [24]. “Under the table” payments emerged in the late 1980s in the former communist countries and as a heritage of the past, they are still common today. In most countries they significantly departed from their original “gratitude” dimension and reached a level of sophistication which make them an institutionalized substitute to a formal co-payment/fee system. This is certainly the case for the Russian Federation where there are no formal co-payments [27].

In summary, most countries offer a relatively comprehensive benefit package, which is identical for the contributing as well as for the subsidized members and sometimes even more comprehensive for the latter. Cost-sharing is widespread, but there are exemptions for specific groups among the exempted.

### Assessment of UHC related performance indicators

#### Population coverage

The share of the total population with (social) health insurance coverage varies widely across countries, as Table 4 shows. Statutory entitlement was lowest in Georgia prior to the reforms (41 % of the population covered by MIP in 2012) [36], and voluntary health insurance for the non-poor [21]. However, total population coverage has increased extremely rapidly within a year to 91 % in 2014 since the introduction of the Universal Health Care Program in early 2013 [36].

**Table 4:** Population coverage

Country	(Social) health insurance coverage of total population	Exempted as share of	
		insured population	eligible population
Albania	less than 50 % (according to household surveys, no year indicated) [37]	n/a	n/a
Bosnia & Herzegovina *a) Federation* ; *b) Republika Srpska*	65 %–83 % (2007) (depending on the part of the country) [80]	50 % (2007) [61]	a) 82 % ; b) 70 % (2007) [81]
Bulgaria	77 % (2011) [38]	35 %^a^ [38]	n/a
Georgia	MIP: 50 % (2012) [21]; Universal Health Care Program: 91 % (2014) [36]	72 % (2010) [53]	73 % (2011) [21]
Kyrgyzstan	78 % (2009) [30]; 85 % of women and 90 % of men (2012) [82]	n/a	n/a
Lithuania	96 % (2008) [83]	58 % (including pensioners)^a^ [83]	n/a
Montenegro	96 % (2008) [84]	unemployed: 25 % (2008) refugees: 3 % (2008) [67]	n/a
Republic of Moldova	72 % (2008); 78 % (2010); 80 % (2011) [39, 49]	65 % (2011) [49]	n/a
Romania	90 % (urban: 95 %; rural: 85 %) (2008) [69]	66 % (including persons in military service)^a^ [43]	n/a
Russian Federation	97 % (2009); 98 % (2010) [27]	n/a	95 % (year not indicated) [27]
Serbia	93 % (2009) [85]	registered unemployed: 2 % (2009) all other exempted groups: 18 % (2009) [86]	n/a
TFYR Macedonia	85 % (2012) [79]	29 % (2012) [79]	n/a
Turkey *(Green Card Scheme)*	95 % (2012) [23]	14 % (2011)^a^ [87]	64 % (2007) [87, 88]

^a^ Calculations by authors based on data from the indicated sources*.*

The enrolment rate is also low for Albania (less than 50 %) [37]. On the other hand, Bosnia & Herzegovina, Bulgaria, Kyrgyzstan, TFYR Macedonia and the Republic of Moldova have higher enrolment rates between 65 and 85 %. The highest share of insured persons with rates between 90 % and nearly 100 % are found in Lithuania, Montenegro, Romania, Serbia, Turkey and the Russian Federation, the latter applying a universalist approach and having the highest enrolment rate of 98 % [38].

Differentiated data on enrolment rates across income quintiles is only available for a few countries and reveals that in Albania and the Republic of Moldova, the poorest part of the population is much less likely to be covered, pointing to inequitable coverage [18, 26, 39]. In contrast, enrolment rates are equal across income quintiles only in Serbia, the much higher total population coverage rate in Serbia also being an explanatory factor [40].

It is equally important to explore the targeting effectiveness of the system, which is the ratio of exempted individuals as of potentially eligible beneficiaries. Although differentiated data is scarce, of those countries with available data, the Russian Federation has the highest percentage of potentially eligible beneficiaries being covered, namely 95 %. In Bosnia & Herzegovina and the Republic of Srpska, this is 82 and 70 % respectively (with most recent data available for 2007). In Georgia, this share reached 73 % (data of 2011).

Another population coverage indicator is the share of the exempted individuals as of the total insured population, which gives an idea about the importance of the exemption arrangement (see Table 4). The greatest shares can be found in Georgia (72 %), Romania (66 %), the Republic of Moldova (65 %) and Lithuania (58 %). In Bosnia & Herzegovina, it is approximately 50 %. The shares in Bulgaria, TFYR Macedonia, Turkey and Serbia are below 35 %.

A final question is whether these financing arrangements succeed to cover all population groups, or whether they fail to cover specific and vulnerable groups. In fact, lack of coverage is a widespread problem among the Roma population in Bosnia & Herzegovina [41], Bulgaria [42], Republic of Moldova [26], Romania [43] and Serbia [40]. The eligibility criteria for exemption are one reason why Roma are less likely to be insured. In themajority of the countries, identity documents, birth certificates or residence permits are required for the enrolment in health insurance schemes. Yet members of the Roma community often do not have such documents [44]. Because of lack of identity documentation and other barriers, a large percentage of the Roma population is not insured. Serbia is the only country which takes this into consideration by explicitly mentioning the “Roma population who due to the traditional way of life do not have permanent living address” as a group eligible for exemption [45], and notably 81 % of the Roma are covered, possibly due to their different enrolment conditions. Although the Roma account only for a small percentage of a country’s total population, the low enrolment rates among them are especially worrisome since they face a higher risk of illness due to their living conditions [24].

Lack of residency and/or identification documents is also a major barrier to cover homeless people or internal migrants in the Russian Federation or TFYR Macedonia [27, 46]. But other groups may also encounter difficulties to benefit from exemption of contributions, such as people in need of social benefits but not entitled to it in Bulgaria [47]. This is because being a beneficiary of social security benefits is often a requirement to be eligible for exemption. For example, in the Republic Moldova unemployment benefits are only granted for six month. Thereafter, the individual also loses health insurance coverage [26]. Such regulations limit coverage in a context of lack of formal employment opportunities.

Likewise, the self-employed and specifically self-employed agricultural workers (farmers) are reported to lack health insurance coverage. In the majority of the countries they have to pay SHI contributions, although at lower rates [48]. At varying incomes, the set contribution rate may turn out to be unaffordable for them such as in Albania [37] or Republic of Moldova [49, 50]. This has implications on enrolment. For example, in the Republic of Moldova, health insurance coverage is compulsory for all citizens with the self-employed having to purchase the policy for themselves. Yet, the penalties for not having insurance coverage have not been fully enforced, making the decision to purchase health insurance in effect a voluntary one [49]. As a consequence, self-employed agricultural workers in the Republic of Moldova are 27 times more likely not to have health insurance compared to formally employed individuals [50]. A survey found that they considered it as too expensive [49]. Low income self-employed farmers are one of the groups where insurance coverage remains challenging [51].

In summary, overall enrolment is relatively high in this region (mostly above 70 %). The exempted make up a large share of the total insured population. The schemes reach at least 70 % of the targeted population. However, even with such arrangements in place, inequalities in insurance coverage between income quintiles persist, and some population groups fall out of the insurance system entirely.

#### Financial protection

In order to analyse the impact of exemption from contributions on financial protection, disaggregated data for the exempted population is needed. However, in the case of integrated schemes, data is usually collected for the insured population as a whole and no distinction is made between the exempted and contributing individuals.

Out-of-pocket (OOP) expenditure as a share of total health expenditure (THE) greatly varies among the studied countries, ranging from 15 % in Turkey up to 62 % in Georgia in the year 2013 (Table 5). Looking over time, in Albania, Bosnia & Herzegovina, Georgia, Kyrgyzstan, TFYR Macedonia and Turkey, OOP as a share of THE decreased. In contrast, in Bulgaria, Lithuania, Montenegro, the Republic of Moldova, Russian Federation and Serbia, OOP as a share of THE increased, which may also be due to increased utilization rates.

**Table 5:** OOP expenditure and access to/utilization of health care services

Country (Year of introduction of government budget transfers)	OOP as % of THE (2013) [15]	Change of OOP as % of THE since the introduction of government revenue transfers [15]	OOP as a share of household expenditure by income quintile (or otherwise indicated)	Utilization of health care services
Albania (1995)	51 %	29 % decrease	2005: 1^st^ quintile: 8 % - 5^th^ quintile: 4 % [18]	n/a
Bosnia & Herzegovina *(Federation (1997) and Republika Srpska (1999))*	29 %	27 % decrease since introduction in Republika Srpska	2004: 1^st^ quintile: 2.3 % - 5^th^ quintile: 1.2 % [18]	n/a
Bulgaria (1998)	40 %	10 % increase	the proportionally highest burden of OOPS falls on the low-and middle income groups [38]	n/a
Georgia *(MIP)*(2006)	62 %	10 % decrease	MIP beneficiaries pay approx. 40–60 % less than non- beneficiaries for outpatient care in the two regions Adjara and Tbilisi and for inpatient care in all regions (2009) [21]	MIP has no impact on utilization rates (data for 2009 and 2010) [21, 89]
Kyrgyzstan (1997)	36 %	11 % decrease	2003: 1^st^ quintile: 7.1 % - 5^th^ quintile: 4.5 % 2009: 1^st^ quintile: 4.4 % - 5^th^ quintile: 4.0 % [30]	utilization of primary care among the poorest quintile increased slightly from 6.3 % in 2001 to 8.1 % in 2009 [30]
Lithuania (1997)	33 %	9 % increase	n/a	n/a
Montenegro (1993)	43 %	13 % increase (since 1995)^a^	2004: 1^st^ quintile: 0.8 % - 5^th^ quintile: 1.1 % [18]	n/a
Republic of Moldova (2004)	45 %	5 % increase	2008: 1^st^ quintile: 2.8 % - 5^th^ quintile: 7.8 % 2009: 1^st^ quintile: 4.1 % - 5^th^ quintile: 11.4 % 2010: 1^st^ quintile: 3.6 % - 5^th^ quintile: 7.4 % [49] The uninsured are 3.8 times more like to have had OOP for outpatient care compared to the insured (in 2012); but nearly 0.5 times for inpatient care [39])	11.2 % of the poorest quintile has consulted a doctor in the past four months compared to 25.5 % of respondents from the richest quintile (2010) Share of exempt population seeking health care in the last 4 weeks: 35.5 % (2008) and 41.7 % (2010) [49]
Romania (1999)	20 %	0 %	n/a	n/a
Russian Federation (1993)	48 %	31 % increase (since 1995)^a^	n/a	People of higher income quintiles consume medical services more frequently than those of lower quintiles, although the latter’s health outcomes are worse (no year indicated) [27]
Serbia (1992)	38 %	12 % increase (since 1995)^a^	2003: 1^st^ quintile: 4.4 % - 5^th^ quintile: 3.6 % [18]	n/a
TFYR Macedonia (1991)	31 %	9 % decrease (since 1995)^a^	n/a	n/a
Turkey *(Green Card Scheme)* (1992)	15 %	15 % decrease (since 1995)^a^	Green Card holders: estimated at 4.1 % (2003), 3.5 % (2006), 4.1 % (2009) [90] OOPs as a share of household consumption expenditure increased for low income groups, but decreased for higher income levels over 2003 to 2006 [91]	Green Card Program associated with a positive and significant impact on protecting health care utilization of the poor during the outbreak of the financial crisis. 22.4 % of the Green Card holders reported forgone use of healthcare services during the last 12 months because of financial barriers, compared to 6.1 % of rest of public insurees [56, 88, 90]

^a^ Introduction of the scheme occurred prior to 1995. National Health Accounts data is available from 1995 onwards

In order to analyse the impact of contribution exemption on financial protection, disaggregated data for the exempted population is needed. However, in the case of integrated schemes, data is usually collected for the insured population as a whole with no distinction made between the exempted and contributing individuals. For that matter, data disaggregated along income quintiles is assessed instead.

Inequalities in OOP as a share of household expenditure between different income quintiles continue to persist, with lower income quintiles having a higher share in most countries, although the gap reduced in Kyrgyzstan. In contrast, in the Republic of Moldova and Montenegro, higher income quintiles have a higher share of OOPs. [30]. Likewise, in Georgia, average OOP payments for inpatient care by the exempted are about 40–60 % of the non-beneficiaries’ OOP expenditure. For outpatient care, OOP for the exempted reduced in those two Georgian regions that cover additional beneficiaries [52].

Data on the incidence of catastrophic expenditure is only available for a few countries (see Additional file 4), with only Georgia and Turkey having disaggregated data for the exempted population. In Turkey, the incidence of catastrophic expenditure for Green Card holders decreased slightly between 2003 and 2009. A much higher share of exempted households experienced catastrophic expenditures in Georgia, i.e.: 22.4 % in 2010. It was also found that (between 2008 and 2010) only 14 % of MIP beneficiaries made use of their insurance coverage during a period when household income decreased and OOP for non-covered pharmaceutical increased due to various exogenous factors. Thus, it has been argued that MIP was only able to cushion the negative impact on the poorest without actually reducing the share of households’ experiencing catastrophic expenditure [53].

The incidence of catastrophic spending reduced for the poorest income quintile in the case of Albania and the Republic of Moldova in recent years. For the latter case, this can be partially explained by the expansion of insurance coverage to the poorest households [49]. Moreover, in Albania, the incidence of impoverishing expenditure also decreased from 6.5 % (2002) to 3.6 % (in 2008) [54]. In contrast, in Serbia, 2007 data reveals that about 6 % of households were impoverished through OOP [18, 55].

In summary, inequalities in OOP expenditure persist despite the existence of government revenue transfers or because coverage rates among the poorest income quintiles are low, with medicines often being a main driver for OOP, such as in the Republic of Moldova [39]. Moreover, despite insurance coverage, people belonging to the bottom income quintile were still the most affected by catastrophic expenditure compared to households at the highest end of the income distribution, as found in Albania, Georgia and Serbia. Altogether, evidence is mixed regarding improvements in financial protection.

#### Access to and utilization of health care services

Data to reveal changes in utilization rates for the exempt is limited. Utilization rates increased for the exempt in Kyrgyzstan and the Republic of Moldova during specific periods, and in the latter case are even higher than for contributors [30, 49]. Yet, there was no improvement for MIP beneficiaries in Georgia [52], and likewise in Turkey [56], the exempt have lower rates than the rest of the insured. Moreover, lower income quintiles have lower utilization rates than higher income quintiles. In summary, it appears that the gap in the utilization rate between richer and poorer income quintiles decreases and rates also have increased for the exempt in some countries, but inequities continue to exist.

## Discussion

This section aims to explore the contribution and the plausible effects of critical institutional design features of such financing arrangements on progress towards UHC. Importantly, there are many other factors, relating to the supply side, service perception, and other developments in the health sector and beyond, which affect and explain UHC progress.

### The type of targeting method chosen

The way eligibility of exempted individuals is defined as well as the targeting method of the exemption arrangements appear to strongly influence enrolment. The data, though scarce, does suggest that enrolment rates are highest with a universalist approach, followed by an indirect targeting approach. The share of the exempted among all insured is larger where indirect targeting is applied compared to direct targeting.

In systems with direct targeting, such as in Turkey and Georgia’s previous MIP, beneficiary identification is more complex since it requires a reliable (proxy) means-test. The effectiveness of means-testing can be assessed by reviewing the number of exempted beneficiaries in the bottom and top income quintile. Even though the population coverage rates of the eligible beneficiaries improved enormously over the years, targeting effectiveness substantially varies across countries. In Turkey, the majority of Green Cards was delivered to the bottom quintile (71 % as per 2008) and 96 % of the poorest quintile were enrolled, with no inclusion error and only a small exclusion error of 6 % [23]. In contrast thereto, Georgia’s MIP identification process faced a relatively high exclusion error, with 54 % of the poorest and 62 % of the second poorest decile being excluded, and some inclusion error, with 5 % of the richest and 8 % of the second richest decile being enrolled in the year 2011.

However, a universalist approach may not necessarily result in 100 % population coverage, as the case of the Russian Federation shows. Moreover, straightforward „going universal“requires significant budget transfers. Countries therefore need to explore whether they have the fiscal space to do so. A possible option for many of these EURO countries with tight fiscal space situations is thus to build upon their targeted approach of focusing on the most needy groups, combined with a clear and well planned time horizon for consecutive scale-ups for expanded coverage.

Moreover, the enrolment procedures are also decisive. Population coverage seems to be enhanced when the authorities are active and in charge of enrolment, rather than the individuals being responsible for their own enrolment. This is because there are various forms of administrative hurdles for potential beneficiaries. For example language barriers prevented minorities in Georgia to apply for MIP [21]. On the other hand, enrolment initiated by the authorities does not automatically imply that individuals know about their insurance status and respective rights.

### Pooling arrangements and defragmentation

In all countries, and in Georgia since 2013 and Turkey since 2012, the exempted individuals are within the same pool as the contributors and their family dependents. Moreover coverage membership is mandatory for both the exempt (except for Turkey) and contributors. These two design aspects enhance equity in access and risk pooling as it and maximizes the redistributive capacity.

In Kyrgyzstan, in order to avoid these problems related to multiple territorial funds, the level of pooling was shifted from the oblast (administrative sub-national division) to the national level in 2006. The desired effects occurred almost immediately after this reform. The inequalities between the oblasts reduced, also translating in more equitable financing at the individual level. OOP as well as utilization rates became more equal across oblasts [20].

Integration of the exempted individuals and striving for universality in health insurance coverage right from the beginning, as most of the EURO countries did, is not common for many LMIC. The typical approach that has been taken by many countries of other regions was to establish a SHI scheme first for formal sector employees and then try to gradually expand coverage to those outside formal sector work over the years [57]. This has proven to be difficult and meant that a large part of the population outside formal sector employment has remained uncovered for a long time (cf. [3, 4, 58]).

### Scope of the benefit package

The comprehensiveness of the benefit package may also influence the incentive to enrol in the SHI scheme, especially when membership is voluntary. For example, in Turkey, the Green Card Scheme’s benefit package initially only covered inpatient health care and the interest to enrol was modest. Outpatient care was added in 2004 and outpatient drugs in 2005. Between 2003 and 2006 the number of registered exempted beneficiaries rose from 4 to 11 % of the population [23], although clearly other factors, e.g. better communication and enrolment outreach, also explain this rise.

The scope of the benefit package also influences the degree of financial protection and utilization rates. In the Republic of Moldova, for example, only a limited set of outpatient drugs is covered [26], which might explain the low utilization rates among the poorest compared to the richest, as well as the low level of financial protection. In Georgia, for example, the MIP with a comprehensive benefit, improved financial protection of its beneficiaries. However, patients still faced OOP, though significantly lower than non-beneficiaries because of the exclusion of outpatient drugs in the benefit package, while utilization rates among beneficiaries did not increase. The insufficient coverage of pharmaceuticals is one possible explanation for this finding since it might demotivate patients from seeking care. Starting in 2010, a small package of outpatient drugs was included [52].

Related to this is the concern about formal co-payments for health services, as they are a barrier for equity in utilization and result in unequitable financial protection in many of the countries assessed. Findings suggest that formal cost-sharing as well as under the table payments significantly reduce utilization rates, with a stronger effect on the poor often belonging to the group with the greatest health needs [26, 27]. In order to enhance equitable financing and access, most countries apply cost-sharing exemptions for some of the non-contributing beneficiaries, notably the most vulnerable groups. For example, in Bosnia & Herzegovina, Serbia and Montenegro, health expenditure as a share of total household expenditure is more similar across quintiles [18], with the cost-sharing exemptions for the non-contributors being possibly one reason.

Finally, a disproportionally higher burden falls on vulnerable and low-income groups due to usually regressive informal payments. Controlling, reducing or turning them into official co-payments remains a challenge for many countries. Causes for informal payments are multiple, and apart from reviewing the benefit package design and the inclusion of medicines, purchasing and provider payment issues also need to be addressed (cf. [39]). Yet, Kyrgyzstan is a positive example where informal payments significantly declined since 2001. It is argued that increased awareness of the patients about the services covered by the benefit package and additional funding for hospitals may have contributed to this [30].

## Conclusion

This paper explored patterns in the institutional design of state budget transfer arrangements to exempt specific, often in particular vulnerable population groups from contributions. It also assessed their plausible impact on deepening UHC, with the aim to identify those key institutional design features being conducive for UHC progress.

Notably, nearly all countries avoided fragmentation and different degrees of benefit package coverage by establishing right from its start a scheme that pooled both contributions and state budget transfers on behalf of the exempted groups, along with mandatory membership for all. Full contribution exemption via general government revenues allowed to cover a large range of population groups outside formal sector work. Exemption from cost-sharing for non-contributing groups is critical. These design features ultimately serve to ensure equity in access and financial protection.

Nonetheless, there remain concerns as to inequity in access and financial protection across income quintiles and population groups. First, there are still a few population groups that are not eligible for exemption, namely the Roma in some countries, needy people without social assistance and self-employed farmers. There is an urgent need to expand eligibility to cover those hard to reach.

Another reform issue is the insufficient coverage of pharmaceuticals, with concerns relating to catastrophic expenditure, impoverishment and informal payments, especially for low-income groups. Finally, the recent financial crisis as well as ongoing demographic changes point to the need to find long-term solutions for the sustainability of such financing arrangements.

Overall, more evidence on the impacts of this financing arrangement should be collected, differentiated across population groups and income quintiles. Further analysis could explore whether policies are effectively being implemented. Evaluations could also assess enrolment proceedings in more detail. For example, is active enrolment by the authorities indeed more conducive to higher enrolment rates than making enrolment the responsibility of the individual?

Altogether, this analysis shows that budget transfers to cover those outside formal sector work can be indeed a viable path to expanding and deepening UHC.

## Additional files

Additional file 1:
**Analytical framework.** Provides a detailed explanation of Table 1 based on which the analytical framework was developed [18, 59, 92–97]. (PDF 58 kb) Additional file 2:
**Benefit package.** Provides information about the health services covered by the benefit package for the exempted groups in each country [21, 23, 24, 26, 27, 30, 31, 34, 36, 38, 44, 46, 65, 67, 68, 70–73, 77, 98–103]. (PDF 69 kb) Additional file 3:
**Cost-sharing mechanisms.** Provides information regarding the cost-sharing mechanism and rates in each country [21, 23, 24, 26, 27, 30, 31, 34, 36, 38, 44, 65, 67, 68, 72, 77, 85, 104–106]. (PDF 71 kb) Additional file 4:
**Incidence of catastrophic and impoverishing expenditure (at 40 % threshold level).** Provides data regarding the incidence of catastrophic and impoverishing health expenditure [18, 49, 53, 55, 74, 90, 107]. (PDF 60 kb)

## Abbreviations

EURO WHO: European Region
GHED: Global health expenditure database
LMIC: Low- and middle-income countries
MIP: Medical Insurance Scheme for the Poor (Georgia)
OOP: Out-of-pocket
SHI: Social health insurance
TFYR Macedonia: The former Yugoslav Republic of Macedonia
THE: Total health expenditure
UHC: Universal health coverage
WHO: World Health Organization

## References

[1] WHO. The World Health Report - Health Systems Financing: the path to universal coverage. Geneva; 2010. 10.2471/BLT.10.078741 PMC2878164 20539847

[2] Meng Q, Yuan B, Jia L, Yu B, Gao J (2010). Expanding health insurance coverage in vulnerable groups: a systematic review of options. Health Policy Plan.

[3] Acharya A, Vellakkal S, Taylor F, Masset E, Satija A, Burke M, et al. Impact of national health insurance for the poor and the informal sector in low-and middle-income countries [Internet]. 2012 [cited 2013 Apr 3]. Available from: http://www.dfid.gov.uk/r4d/PDF/Outputs/SystematicReviews/Health-insurance-2012Acharya-report.pdf

[4] Bitran R. Universal health coverage and the challenge of informal employment: Lessons from developing countries. Washington (DC); 2014.

[5] Tangcharoensathien V, Patcharanarumol W, Ir P, Aljunid SM, Mukti AG, Akkahavong K (2011). Health-financing reforms in southeast Asia: challenges in achieving universal coverage. Lancet.

[6] Davis C. Understanding the legacy: health financing systems in the USSR and central and eastern Europe prior to transition. In: Kutzin et al., editor. Implementing Health Financing Reform: Lessons from countries in transition. Geneva; 2010. p. 25–64.

[7] WHO Regional Office for Europe. WHO/Europe | Home [Internet]. 2016 [cited 2016 Jan 10]. Available from: http://www.euro.who.int/en/home

[8] Kutzin J, Cashin C, Jakab M, editors. Implementing Health Financing Reform: Lessons from countries in transition. Geneva; 2010.

[9] Kutzin J, Jakab M, Cashin C (2010). Lessons from health financing reform in central and eastern Europe and the former Soviet Union. Health Econ Policy Law.

[10] Vilcu I, Mathauer I. State Budget Transfers to Health Insurance Funds for Universal Health Coverage: Institutional design patterns and challenges of covering those outside the formal sector in Eastern European high-income countries. J Equity Health. 2016 10.1186/s12939-016-0295-y PMC4714511 26767970

[11] Vilcu I, Probst L, Bayarsaikhan D, Mathauer I. Government subsidization of health insurance type arrangements for vulnerable and informally employed population groups in Asia: Trends in institutional design. Geneva; 2016.

[12] Mathauer I, Behrendt T. Subsidization of Health Insurance Coverage for Vulnerable Groups in Latin America: Trends in Institutional Design and Challenges. Geneva; 2016.

[13] Blumenthal D, Mort E, Edwards J (1995). The efficacy of primary care for vulnerable population groups. Health Serv Res.

[14] The World Bank. World Bank country classification [Internet]. [cited 2015 Jul 14]. Available from: http://data.worldbank.org/about/country-classifications

[15] WHO. Global Health Expenditure Database [Internet]. 2015. Available from: http://apps.who.int/nha/database

[16] Katsaga A, Kulzhanov M, Karanikolos M, Rechel B (2012). Kazakhkstan: Health system review. Health Syst Transit.

[17] Kutzin J (2001). A descriptive framework for country-level analysis of health care financing arrangements. Health Policy.

[18] Bredenkamp C, Mendola M, Gragnolati M (2011). Catastrophic and impoverishing effects of health expenditure: new evidence from the Western Balkans. Health Policy Plan.

[19] Preker A, Jakab M, Schneider M, Mossialos E, Dixon A, Figueras J, Kutzin J (2002). Health financing reforms in central and eastern Europe and the former Soviet Union. Funding health care: options for Europe.

[20] Kutzin J, Jakab M, Shishkin S (2009). From scheme to system: social health insurance funds and the transformation of health financing in Kyrgyzstan and Moldova. Adv Health Econ Health Serv Res.

[21] Smith O (2013). Georgia’s medical insurance program for the poor.

[22] WHO. Georgia: Country Cooperation Strategy [Internet]. Georgia: Country Cooperation Strategy. 2013 [cited 2014 Jun 22]. Available from: http://www.who.int/countryfocus/cooperation_strategy/briefs/en/

[23] Mollahaliloglu S, Atun R, Postolovska I (2013). Toward universal coverage: Turkey’s green card program for the poor.

[24] Vlădescu C, Scintee G, Olsavszky V, Allin S, Mladovsky P. Romania: Health System Review. Health Care Syst Transit. 2008;3(10):1–172. 27603897

[25] Mathivet B (2007). La réforme des systèmes de santé dans les pays en transition: le cas de la Russie. Etat Economie Appliquée.

[26] Turcano G, Domente S, Buga M, Richardson E (2012). Republic of Moldova: Health System Review. Health Care Syst Transit.

[27] Popovich L, Potapchik E, Shishkin S, Richardson E, Vacroux A, Mathivet B (2011). Russian Federation: Health System Review. Health Care Syst Transit.

[28] Kutzin J. Conceptual framework for analysing health financing systems and the effects of reforms. In: Kutzin et al., editor. Implementing Health Financing Reform: Lessons from countries in transition. 2010.

[29] Tragakes E, Lessof S. Russian Federation: Health System Review. European Observatory on Health Systems and Policies, editor. Health Care Systems in Transition. 2003;5(3).

[30] Ibraimova A, Akkazieva B, Ibraimov A, Manzhieva E, Rechel B (2011). Kyrgyzstan: Health system review. Health Syst Transit.

[31] Kurti S (2011). Albania: Health Care System in the Course of Health Reform. An Overview of Health Insurance System. Mediterranean J Soc Sci.

[32] Gotsadze G, Gaál P. Coverage decisions: benefit entitlements and patient cost sharing. In: Kutzin J, Cashin C, Jakab M, editors. Implementing Health Financing Reform: Lessons from countries in transition. Geneva; 2010. p. 187–218.

[33] Robinson R, Mossialos E, Dixon A, Figueras J, Kutzin J (2002). User charges for health care. Funding health care: options for Europe.

[34] Cain J, Duran A, Fortis A, Jakubowski E. Health Care Systems in Transition: Bosnia and Herzegovina. Cain, J., Jakubowski, E., editors. 2002;4(7).

[35] The World Bank (2011). Romania - Functional review: health sector.

[36] WHO, The World Bank, USAID (2014). A Review of Universal Health Coverage Reforms Introduced in Georgia since February 2013.

[37] Imasheva A, Seiter A. The Pharmaceutical Sector of the Western Balkan Countries. The World Bank. 2008.

[38] Dimova A, Rohova M, Moutafova E, Atanasova E, Koeva S, Panteli D, et al. Bulgaria: Health System Review. Health Care Systems in Transition. 2012;3. 22894828

[39] Vian T, Feeley FG, Domente S, Negruta A, Matei A, Habicht J (2015). Barriers to universal health coverage in Republic of Moldova: a policy analysis of formal and informal out-of-pocket payments. BMC Health Serv Res.

[40] Idzerda L, Adams O, Patrick J, Schrecker T, Tugwell P (2011). Access to primary healthcare services for the Roma population in Serbia: a secondary data analysis. BMC Int Health Human Rights.

[41] Colombini M, Rechel B, Mayhew S (2012). Access of Roma to sexual and reproductive health services: Qualitative findings from Albania, Bulgaria and Macedonia. Glob Public Health.

[42] Atanasova E, Pavlova M, Moutafova E, Rechel B, Groot W. Out-of-pocket payments for health care services in Bulgaria: financial burden and barrier to access. Eur J Public Health. 2012;1–7. 10.1093/eurpub/cks169 23220626

[43] Anton S, Gavrilovici C, Oprea L (2013). Ethical Issues in Financing Health Care in Romania. Soc Res Rep.

[44] Nuri B, Tragakes E (2002). Albania. Health Care Systems in Transition.

[45] Republic of Serbia (2005). Health Insurance Act.

[46] Uzunov V. Socio-Economic Transformation and the Welfare System of the Republic of Macedonia in the Period of Transition. In: Friedrich Ebert Stiftung, editor. Welfare States in Transition - 20 Years after the Yugoslav Welfare Model. Sofia, Bulgaria; 2011. p. 115–34.

[47] Extending Social Security to All: A guide through challenges and options. Geneva; 2010.

[48] Sheiman I, Lagenbrunner J, Kehler J, Cashin C, Kutzin J. Sources of funds and revenue collection: reforms and challenges. In: Kutzin J, Cashin C, Jakab M, editors. Implementing Health Financing Reform: Lessons from countries in transition. Geneva; 2010. p. 87–118.

[49] Shishkin S, Jowett M (2012). A Review of Health Financing Reforms in the Republic of Moldova.

[50] Richardson E, Roberts B, Sava V, Menon R, McKee M (2012). Health insurance coverage and health care access in Moldova. Health Policy Plan.

[51] Mills A, Bennett S, Mossialos E, Dixon A, Figueras J, Kutzin J (2002). Lessons on the sustainability of health care funding from low- and middle-income countries. Funding Health Care: Options for Europe.

[52] Bauhoff S, Hotchkiss D, Smith O (2011). The impact of medical insurance for the poor in Georgia: a regression discontinuity approach. Health Econ.

[53] Zoidze A, Rukhazde N, Chkhatarashvili K, Gotsadze G (2013). Promoting universal financial protection: health insurance for the poor in Georgia – a case study. Health Res Pol Syst.

[54] Tomini SM, Packard TG, Tomini F (2013). Catastrophic and impoverishing effects of out-of-pocket payments for health care in Albania: evidence from Albania Living Standards Measurement Surveys 2002, 2005 and 2008. Health Policy Plan.

[55] Arsenijevic J, Pavlova M, Groot W (2013). Measuring the catastrophic and impoverishing effect of household health care spending in Serbia. Soc Sci Med.

[56] Aran MA, Hentschel JS. Protection in Good and Bad Times? The Turkey Green Card Health Program. The World Bank. 2012.

[57] Savedoff WD (2004). Is there a case for social insurance?. Health Policy Plan.

[58] González Rossetti A (2002). Social health insurance in Latin America. Background paper prepared for the DFID Health Insurance Workshop.

[59] Xu, K (2005). Distribution of health payments and catastrophic expenditures: Methodology.

[60] Tavanxhi N, Burazeri G, Laaser U, Rademacher R (2006). Financing Health Care in Albania, Trends, Major Challenges and the Way Forward. Financing Health Care – A Dialogue between South Eastern Europe and Germany.

[61] Bredenkamp C, Gragnolatti M. Sustainability of Healthcare Financing in the Western Balkans: An Overview of Progress and Challenges. The World Bank, editor. World Bank Publications; 2007.

[62] Meimanaliev A, Ibraimov A, Elebesov B, Rechel B. Health Care Systems in Transition: Kyrgyzstan. 2005;7(2).

[63] Giuffrida A, Jakab M, Dale E (2013). Toward Universal Coverage in Health: The Case of the State Guaranteed Benefit Package of the Kyrgyz Republic.

[64] WHO. Country Cooperation Strategy at a glance - Lithuania [Internet]. 2011. Available from: http://www.who.int/countryfocus/cooperation_strategy/ccsbrief_ltu_en.pdf

[65] Cuadra C (2010). Policies on Health Care for Undocumented Migrants in EU27- Country Report Lithuania.

[66] Jankauskiene D, Medaiskis T (2012). Annual National Report Lithuania 2012- Pensions, Health Care and Long-Term Care.

[67] Vuković D, Perivsić N. Opportunities and Challenges of Social Security Transition in Montenegro. In: Dehnert S, editor. Welfare States in Transition: 20 Years after the Yugoslav Welfare Model. Sofia, Bulgaria; 2011. p. 165–201.

[68] Republic of Montenegro (2004). The Law on Health Insurance.

[69] Predescu M (2008). Quality in and Equality of Access to Healthcare Services: Country Report for Romania.

[70] Gavrilović A, Trmčić S (2013). Health Insurance System in Serbia- Quality.

[71] Gajic-Stevanovic M. Health care system and spending in Serbia. Public Health Institute of Serbia; 2009.

[72] Djikanovic B, Laaser U, Rademacher R, Health Care Financing in Serbia (2006). Financing Health Care – A Dialogue between South Eastern Europe and Germany.

[73] Donev D. Health Insurance System and Financing of Health Care in the Republic of Macedonia. In: Laaser, U., Rademacher, R., editors. Financing Health Care – A Dialogue between South Eastern Europe and Germany [Internet]. Eschborn, Bonn: German Organisation for Technical Cooperation (GTZ) and Federal Ministry for Economic Cooperation and Development (BMZ); 2006 [cited 2013 May 22]. p. 133–54. Available from: http://www.researchgate.net/publication/233905760_Financing_Health_Care_-_A_Dialogue_between_South_Eastern_Europe_and_Germany/file/32bfe5109b2cf0d713.pdf#page=129

[74] Özgen NH, Şahin İ, Yıldırım HH (2015). Financial catastrophe and poverty impacts of out-of-pocket health payments in Turkey. Eur J Health Econ.

[75] Jankauskiene D. Management of Economic and Financial Crisis in Health Care Sector in Lithuania. Manag Health. 2010;(XIV/4/2010):3–10.

[76] Murauskiene L, Janoniene R, Veniute M, van Ginneken E, Karanikolos M (2013). Lithuania: Health System Review. Health Syst Transit.

[77] Ministry of Health of Lithuania. Patients Fund: Overview 2007. Vilnius; 2011.

[78] OECD, Eurostat, WHO. A System of Health Accounts [Internet]. OECD Publishing; 2011 [cited 2015 Feb 8]. Available from: http://www.oecd-ilibrary.org/social-issues-migration-health/a-system-of-health-accounts_9789264116016-en

[79] Health Insurance Fund of Macedonia. Insured Persons [Internet]. Health Insurance Fund of Macedonia. 2012 [cited 2013 Jun 8]. Available from: http://www.fzo.org.mk/default-en.asp

[80] WHO (2007). Country Cooperation Strategy at a Glance - Bosnia & Herzegovina.

[81] Schneider P. Implementation Completion and Results Report to Bosnia and Herzegovina for a Social Insurance Technical Assistance Project. The World Bank; 2008 p. 1–52. Report No.: ICR0000733.

[82] National Statistical Committee of the Kyrgyz Republic, Ministry of Health, ICF International (2013). Kyrgyz Republic Demographic and Health Survey 2012.

[83] Kacevicius G (2010). Mandatory Health Insurance system in Lithuania: an overview.

[84] Jelusic B. National Human Development Report 2013: “People are the Real Wealth of the Country.” How Rich is Montenegro? UNDP. 2013.

[85] Vuković D, Perivsić N. Social Security in Serbia - Twenty Years Later. In: Stambolieva M, Dehnert S, editors. Welfare States in Transition: 20 Years after the Yugoslav Welfare Model. Sofia, Bulgaria; 2011. p. 228–61.

[86] Republic Fund of Health Insurance Serbia. Republic Institute For Health Insurance - Number of insurees [Internet]. Number of Insurees. 2009 [cited 2013 May 24]. Available from: http://www.eng.rfzo.rs/index.php/number-of-insurees

[87] Erus B, Yakut-Cakar B, Cali S, Adaman F (2015). Health policy for the poor: an exploration on the take-up of means-tested health benefits in Turkey. Soc Sci Med.

[88] Atun R, Aydın S, Chakraborty S, Sümer S, Aran M, Gürol I (2013). Universal health coverage in Turkey: enhancement of equity. Lancet.

[89] Gotsadze G, Zoidze A, Rukhadze N, Shengelia N, Chkhaidze N (2015). An impact evaluation of medical insurance for poor in Georgia: preliminary results and policy implications. Health Policy Plan.

[90] Yardim MS, Cilingiroglu N, Yardim N (2014). Financial protection in health in Turkey: the effects of the Health Transformation Programme. Health Policy Plan.

[91] Ökem ZG, Çakar M (2015). What have health care reforms achieved in Turkey? An appraisal of the “Health Transformation Programme.”. Health Policy.

[92] Wang H, Switlick K, Ortiz C, Connor C, Zurita B. Health Insurance Handbook- How To Make It Work. Connor C, Wang H, editors. Abt Associates Inc.; 2010.

[93] Dixon A, Langenbrunner J, Mossialos E. Facing the challenges of health care financing. In: Figueras et al., editor. Health Systems in Transition: learning from experience. Copenhagen; 2004. p. 51–84.

[94] Witter S (2002). Health financing in developing and transitional countries. Briefing Paper for OXFAM, University of York, York.

[95] WHO. WHO | Universal coverage - three dimensions [Internet]. Health Financing for Universal Coverage. 2013 [cited 2013 Jul 11]. Available from: http://www.who.int/health_financing/strategy/dimensions/en/

[96] Tragakes E, Brigis G, Karaskevica J, Rurane A, Stuburs A, Zusmane E (2008). Latvia: Health System Review. Health Syst Transit.

[97] WHO, The World Bank (2013). Monitoring Progress towards Universal Health Coverage at Country and Global Levels: A Framework.

[98] Maglajlić A, Rašidagić K. Socio-Economic Transformation in Bosnia and Herzegovina. In: Stambolieva M, Dehnert S, editors. Welfare States in Transition: 20 Years after the Yugoslav Welfare Model. Sofia, Bulgaria; 2011. p. pp. 16–40.

[99] Chanturidze T, Ugulava T, Durán A, Richardson E (2009). Georgia: Health System Review. Health Syst Transit.

[100] Europe-cities. Health in Lithuania. Healthcare system of Lithuania [Internet]. Healthcare in Lithuania. [cited 2013 Feb 4]. Available from: http://www.europe-cities.com/en/633/lithuania/health/

[101] CEED Consulting (2011). Integrity Assessment of the Health Care System in Montenegro. Podgorica.

[102] Project for Improvement of Good Governance in Montegrin Healthcare System. Podgorica; 2011.

[103] Tatar M, Mollahaliloglu S, Sahin B, Aydin S, Maresso R, Hernández-Quevedo C (2011). Turkey: Health System Review. Health Syst Transit.

[104] The World Bank. Albania Health Sector Note, Report No. 32612-AL [Internet]. 2006. Available from: http://web.worldbank.org/archive/website01337/WEB/IMAGES/32612.PDF

[105] IMF (2004). Bosnia and Herzegovina: poverty reduction strategy paper- mid-term development strategy.

[106] Health Insurance Fund of Macedonia (2012). Annual report 2011.

[107] Scheil-Adlung X, Kuhl C. Addressing inequities in access to health care for vulnerable groups in countries of Europe and Central Asia. Geneva; 2011. Report No.: Social Security Policy Briefings: Paper 8.

