# Supplementary material for: State budget transfers to health insurance funds: extending universal health coverage in low- and middle-income countries of the WHO European Region
Source: Int J Equity Health. 2016 Apr 2;15:57. doi: 10.1186/s12939-016-0321-0 (PMC4818884; doi:10.1186/s12939-016-0321-0)
Supplement: Additional file 1: — Analytical framework. Provides a detailed explanation of Table 1 based on which the analytical framework was developed [18, 59, 92–97]. (PDF 58 kb) [file 12939_2016_321_MOESM1_ESM.pdf]

Additional File 1. Analytical framework

Institutional design features of budget transfer/subsidization arrangements

Eligibility and enrolment rules

The eligibility rules specify who is eligible to benefit from exemption of paying health insurance contribution (92). The eligibility rules reflect a country's understanding of which population groups are considered "vulnerable" with regard to health care access. In order to determine who is eligible various identification methods can be employed. If eligibility is "universalist" no population group is targeted specifically. Instead all individuals fulfilling a very broad criterion are eligible (e.g. the entire population not insured in any other formal sector insurance scheme, or all residents of a country). The alternatives are direct or indirect targeting. In case of "direct" targeting eligible individuals are identified through a means assessment (e.g. means testing, proxy means testing) (92). In contrast, "indirect" targeting focuses on broader, usually observable geographical, demographic and/or socio-economic characteristics supposedly correlated with poverty and vulnerability. Finally, membership of the exempted individuals can be either mandatory or voluntary (92). All of these institutional design aspects influence the population coverage rate of the exempted.

Financing arrangements

One important institutional design aspect is whether exemption from contributions for the eligible groups is complete or partial, which is decisive for the enrolment rate. In

case of a partial exemption the remaining part needs to be paid by the individual. This may be especially difficult for low-income groups and prevent them from enrolling.

Contributions on behalf of the exempted can be financed from a number of different sources, i.e. general government revenues (from central/regional/local levels) or social security funds, each of which may create specific incentives or disincentives for the responsible authorities in charge of enrolling eligible individuals, which again affects enrolment rates. Another important aspect is the way the total amount allocated for the exempted is determined. This may be a set or negotiated lump sum for the entire exempt population or based on a per capita formula logic for each exempt.

Pooling arrangements

Pooling is defined as the accumulation of pre-paid revenues on behalf of the population (93). The way resources are pooled determines how the costs of illness are shared between individuals (risk pooling) and the scope for cross-subsidization (94). In this context, we assess whether the system is one of a single pool or of multiple pools and whether the exempted population groups are part of the same scheme as the contributors or whether they belong to a separate fund. These institutional design features influence financial protection as well as the level of equity in financing and access and the efficiency of the SHI system.

Purchasing

In generic terms, purchasing is defined as the allocation of pooled funds to the health care service providers in order to buy specific services. An important aspect of purchasing is the set of provider payment mechanisms. What is of relevance here is whether payment mechanisms and rates paid to providers are the same for the exempt

and contributors, and if not which implications this has. Another key design feature of purchasing is the benefit package, i.e. the range of services covered as well as the related cost-sharing mechanisms and rates. A benefit package can be considered as fairly comprehensive when it includes primary care, specialized outpatient care and inpatient care as well as essential medicines. A more limited package and the cost-sharing schedule (cost-sharing rates, cost-sharing ceilings or benefit ceilings) affect utilization rates and hence the level of equity in access as well as the extent of financial protection.

UHC related performance indicators

Progress towards UHC will be assessed with regards to improvements over time in the three universal coverage dimensions, namely 1) population coverage, 2) financial protection and 3) access to and utilization of needed health care services (95). These three dimensions are closely linked, and to assess progress towards UHC, it is important to consider them jointly.

Population coverage

Health insurance population coverage will be assessed in two ways. First, total population coverage indicates the share of the population covered by (social) health insurance. Additionally, coverage rates by income quintile provide insight into the equity of coverage. Second, the share of the exempted population as a ratio of the entitled group reveals the targeting effectiveness. Finally, as a ratio of the total population and the insured population, it gives an idea of the magnitude of such financing arrangement.

Financial protection

There are several and complementary ways to measure financial protection depending on data availability. A starting point is to assess trends in out-of-pocket (OOP) expenditure as a share of total health expenditure (THE). Second, OOP as a share of household expenditure can be explored. Thirdly, financial protection can be measured by the incidence of “catastrophic expenditure”. As per the WHO methodology used, it occurs “when a household’s total out-of-pocket health payments equal or exceed 40% of household’s non-subsistence expenditure (…).” (59). As such, it indicates “the extent to which households are protected against income loss due to health expenditures” (18). Another measure is the headcount of households with impoverishing health expenditure, or data on its depth, but data to compare across years or across different groups was not available. Differentiated data along income quintiles or between the insured/uninsured and exempted/contributing population helps to reveal potential inequities.

Access and utilization to needed health services

The financial protection indicators do not reveal whether people are prevented from accessing health care (96). There is thus the need to look at whether people have effective coverage of needed health services (97). This requires data on health care needs. For outpatient and inpatient care services, which is the focus of the benefit packages of the arrangements under study here, this data may be less readily available. For that matter, we use utilization rates data for inpatient and outpatient care as proxy indicators; again at best this is differentiated along income quintiles or between exempted and contributing groups in order to reveal potential inequities in the use of services.

Additional File 2. Benefit package

Country	Services covered for the exempted population groups	Scope of services for the exempt compared to contributors
Albania	only complementary to state-guaranteed benefit package (which offers specialized outpatient and inpatient care at public facilities free of charge for the entire population regardless of insurance status): basic services like family doctor's services, free visit to general practitioner, 90-100% of the cost of 12 tertiary unique examinations, primary and inpatient care in the city of Durres (pilot project since 2001)<br>dental: only emergency care, but broader package for patients < 18 years<br>pharmaceuticals: 410 drugs of the Essential Drug List fully/partially covered (44) (31)	more comprehensive: broader dental care package for patients < 18 years (44)
Bosnia & Herzegovina – *Federation*	comprehensive: primary care, specialized outpatient care, inpatient care<br>pharmaceuticals: drugs of the Essential Drug List covered (approx. 220 drugs, but number varies across cantons)<br>dental: n/a<br>other: refund of travel expenses incurred while seeking medical care (98) (34)	same (97) (34)
Bosnia & Herzegovina – *Republika Srpska*	information about the comprehensiveness of the benefit package n/a<br>pharmaceuticals: inpatient drugs, outpatient drugs of the Essential Drug List covered (220 drugs) (26)<br>dental: n/a	same (34)
Bulgaria	comprehensive: primary care, specialized outpatient care, inpatient care<br>dental: dental care partially covered<br>pharmaceuticals: inpatient and outpatient drugs on Positive Drug List and for certain disease fully/ partially covered (38)	same (38)
Georgia *(MIP)*	comprehensive: primary care, specialized outpatient care, inpatient care<br>pharmaceuticals: inpatient pharmaceuticals, outpatient pharmaceuticals partially covered up to 30 US$ annually (since 2010), drugs for some conditions (diabetes, Tb, HIV/AIDS) provided by the state without charges<br>dental care: not covered (21) (99)	Some differences in benefits packages and co-payments, but these are generally pro-poor (60)
Kyrgyzstan	only complementary to state- guaranteed benefit package (which offers emergency and primary care free of charge for the entire population)<br>the insured are exempt from co-payments to 60-66% of the average cost of treatment in hospitals, and to 50% of diagnostic costs in outpatient facilities; outpatient drugs from the Additional Drug Package covered (424 drugs in 2008) (30)	Same (30)
Lithuania	comprehensive: primary care, specialized outpatient care, inpatient care<br>dental: check-ups are free, but patients must pay for any work done<br>pharmaceuticals: drugs under the List of Compensated Medicines partially/ fully covered, full coverage of medication for chronic diseases that are prevalent among elderly people and medicines for grave diseases (cancer, TB, asthma, etc.) (77) (100)	same (32) (50)
Montenegro	comprehensive: primary care, specialized outpatient care, inpatient care<br>dental: only routine visits and preventive check-ups<br>pharmaceutical: drugs of the Positive Drug List covered<br>other: refund of travel expenses incurred while seeking medical care (67) (101) (102) (68)	same (67) (68)

Country	Services covered for the exempted population groups	Scope of services for the exempt compared to contributors
Republic of Moldova	complementary to state-guaranteed benefit package (which offers emergency and primary care free of charge regardless of insurance status, TB, HIV and mental health also provided free of charge): specialized outpatient care, inpatient care<br>dental: some services (mostly emergency care and preventive check-ups), additional dental care for children < 18 years and pregnant women<br>pharmaceuticals: limited range of outpatient pharmaceuticals fully/ partially covered (38 medicines for the treatment of common conditions) (26)	more comprehensive: additional dental care for children < 18 years and pregnant women (26)
Romania	comprehensive: primary care, specialized outpatient care, inpatient care<br>dental: preventive services and 88 additional procedures fully/ partially covered<br>pharmaceuticals: outpatient drugs on Positive Drug List fully/ partially covered (24)	same (24)
Russian Federation	comprehensive ("daily needs care"):<br>primary care, specialized outpatient care, inpatient care<br>dental: some services only for children, war veterans, and other special groups<br>pharmaceuticals: inpatient pharmaceuticals; outpatient pharmaceuticals only covered<br>for children < 3 years, children from large families < 6 years, disabled people, citizens affected by radiation in the Chernobyl disaster, certain medical conditions, retired individuals receiving minimum pensions, parents and wives of deceased military serviceman, war veterans (11% of population in 2008) (27)	more comprehensive: dental care and outpatient pharmaceuticals also covered for some groups within the exempt population (27)
Serbia	comprehensive: primary care, specialized outpatient care, inpatient care<br>dental: some services only covered only for children, people > 65 years, pregnant women, and in emergency cases<br>pharmaceuticals: inpatient drugs, outpatient prescription drugs covered (71) (70) (724)	more comprehensive: dental care for children, elderly > 65 years, and pregnant women (71)
TFYR Macedonia	comprehensive: primary care, specialized outpatient care, inpatient care<br>pharmaceuticals: inpatient and outpatient drugs in accordance to the List of Medicines covered<br>dental: some dental care<br>other: refund of travel expenses incurred while seeking medical care (46) (73)	more comprehensive: also medical rehabilitation of certain chronic non-communicable diseases for children < 18 years (73)
Turkey *(Green Card Scheme)*	comprehensive: primary care, specialized outpatient care, inpatient care<br>but only covered for services received in public facilities; exempted patients can only use private facilities in emergency cases or if public facilities are fully occupied<br>dental: inpatient and outpatient dental care (broad range of services)<br>pharmaceuticals: inpatient drugs, outpatient prescription drugs partially covered (103) (23)	same (23)

Additional File 3. Cost-sharing mechanisms

Country	Cost-sharing mechanism and rates for all members	Same/different compared to contributing insured population
Albania	Co-payments for outpatient services (insurance contributions in theory cover approx. 50% of service costs); co-insurance for pharmaceuticals (31)(104)	Different (less):<br>Infants < 1 year, invalids, and war veterans exempt from co-insurance for pharmaceuticals (44)
Bosnia & Herzegovina<br>*– Federation*	Co-payments for services (34)	Different (less):<br>Co-payments depend on the patient's social status and available resources (34)(105)
Bosnia & Herzegovina<br>*– Republika Srpska*	Cost-sharing for services (mechanism not specified) (34)	n/a
Bulgaria	User fees for services: 1% of the minimum monthly salary per outpatient visit and 2% of the minimum monthly salary per day of hospitalization (up to 10 bed-days per year),<br>Co-insurance for outpatient pharmaceuticals (38)	Different (less):<br>There is complete exemption from cost-sharing for children, pregnant women, individuals with income below a certain threshold, chronically sick patients, the unemployed, and some other groups (but no exemptions for laboratory tests and outpatient pharmaceuticals); Children deprived of parental care, prisoners, children < 18 years with mental disorders exempt from user fees for dental care (38)
Georgia<br>*(MIP)*	Benefit ceilings for defined services (e.g., 9,000 US$ for an operation); co-insurance for outpatient pharmaceuticals: 50% of the costs (21)	n/a
Kyrgyzstan	Co-insurance for services: 50% of the costs of all non-basic outpatient diagnostic tests (basic test defined as the most frequent 10 tests), 34-40% of the average costs of inpatient treatment (30)	Different (less):<br>Cost-sharing varies depending on exemption category, region, and disease profile (30)
Lithuania	Co-payments for dental care for adults,<br>Co-insurance for outpatient pharmaceuticals (66)	Different (less):<br>Various groups including disabled persons, people who are only partially able to work, people receiving social pensions are refunded 50% of the base price* of the refundable medicines;<br>Children < 18 years and persons recognized as unable to work exempt from co-insurance for outpatient pharmaceuticals (77)

Country	Cost-sharing mechanism and rates for all members	Same/different compared to contributing insured population
Montenegro	Co-payments for services: vary depending on the type of disease, diagnostic, treatment, and rehabilitation cost as well as health care level (67)	Different (less):<br>Children, pregnant women, women during delivery and one year after, social welfare beneficiaries as well as people with certain disease exempt from co-payments (68)(67)
Republic of Moldova	Co-insurance for pharmaceuticals (26)	Different (less):<br>Children < 18 years, pregnant women, diabetes patients exempt from co-insurance (26)
Romania	Co-payments for services: for long-stay care as well as some ambulatory services;<br>Co-insurance for some categories of pharmaceuticals: 10% or 50% of the reference price (24)	Different (less):<br>Pregnant women, postpartum mothers, and children exempt from co-insurance and co-payments (irrespective of their insurance status),<br>Children < 18 years and youths < 26 years if enrolled in any form of education exempt from co-payments for dental care (24)
Russian Federation	No cost-sharing, other than for outpatient pharmaceutical (27)	Same (27)<br>Specific population groups are exempted from fees for outpatient pharmaceuticals
Serbia	Co-insurance for services: up to 35% of the prices of health care services,<br>Co-insurance for pharmaceuticals (72)	Different (less):<br>Pregnant women, disabled people, the unemployed, material assistance recipients, elderly > 65 years are exempt from cost-sharing (85)
TFYR Macedonia	Co-payments for services (except emergency and primary care),<br>Co-insurance for pharmaceuticals; The maximum payment amount for total cost-sharing is 70% from the average net salary of the previous year (106)	Different (less):<br>The maximum payment amounts for total cost-sharing are 20%-40% from the average net salary of the previous year for the persons with lower incomes, children, and the elderly > 65 years,<br>Recipients of social assistance; persons placed in an institution for social protection or in another family, except for medicines prescribed at the PHC level and for the treatment abroad; persons with mental diseases in the psychiatry hospital and the mentally retarded persons without parent care; children with special needs; infants < 1 year; war-disabled persons and their families exempt from cost-sharing (106)
Turkey<br>*(Green Card Scheme)*	Co-payments for services: 4.8 US$ for outpatient visits with a discount of 1.8 US$ if no medicine is prescribed,<br>Co-insurance for outpatient pharmaceuticals: 20% of the costs (for retirees: 10%) (23)	Different (more):<br>Also co-payments for dental care, prosthetics, and orthotics (23)

** The base price for compensated medicines is set every year by the Ministry of Health and might differ from the actual retail price. The base price is reimbursed to a certain extent, whereas the difference between the base price and the retail price needs to be paid OOP.*

Additional File 4. Incidence of catastrophic and impoverishing expenditure (at 40% threshold level)

Country	Catastrophic expenditure				Incidence of impoverishing expenditure
	Total population	Poorest quintile	Richest quintile	Only the exempted	
Albania (54) *at 25% threshold level*	8.1 (2002)<br>6.0 (2005)<br>5.4 (2008)	12.2 (2002)<br>13.2 (2005)<br>10.0 (2008)	4.1 (2002)<br>1.7 (2005)<br>2.9 (2008)	n/a	Increased by 6.5 pp in 2002, by 4.3 pp in 2005, by 3.6 pp in 2008
Bosnia & Herzegovina (18)	n/a	n/a	n/a	n/a	3% (2004)
Bulgaria	n/a	n/a	n/a	n/a	n/a
Georgia (52)	n/a	17.7 (2007)<br>27.0 (2010)	10.3 (2007)<br>20.6 (2010)	22.4 (2010)	n/a
Kyrgyzstan (107)	0.6 (2009)	n/a	n/a	n/a	n/a
Lithuania (107)	1.4 (2009)	n/a	n/a	n/a	n/a
Montenegro (18)	n/a	n/a	n/a	n/a	0.4 (2004)
Republic of Moldova (49)	n/a	4.1 (2007)<br>6.0 (2008)<br>3.1 (2010)<br>3.1 (2011)	3.2 (2007)<br>4.3 (2008)<br>6.4 (2010)<br>5.1 (2011)	n/a	n/a
Romania (107)	0.1 (2009)	n/a	n/a	n/a	n/a
Russian Federation (107)	6.0 (2009)	n/a	n/a	n/a	n/a
Serbia (107) (55)	2.3 (2007)<br>2.4 (2009)	5.6 (2007)	0.5 (2007)	n/a	1.2% (2003)<br>5.9% (2007)
TFYR Macedonia	n/a	n/a	n/a	n/a	n/a
Turkey (90)(74)	below 1%, but share of households increased from 2004-2010	Increased risk	Lower risk	1.7 (2003)<br>0.9 (2006)<br>0.9 (2009)	1.2% (2003)<br>2.2% (2006)<br>1.0% (2009)

Additional Files

Additional File 1. Portable Document Format (.pdf). **Analytical framework**

Provides a detailed explanation of Table 1 based on which the analytical framework was developed.

Additional File 2. Portable Document Format (.pdf). **Benefit package**

Provides information about the health services covered by the benefit package for the exempted groups in each country.

Additional File 3. Portable Document Format (.pdf). **Cost-sharing mechanisms**

Provides information regarding the cost-sharing mechanism and rates in each country.

Additional File 4. Portable Document Format (.pdf). **Incidence of catastrophic and impoverishing expenditure (at 40% threshold level)**

Provides data regarding the incidence of catastrophic and impoverishing health expenditure.
