# Supplementary material for: State budget transfers to health insurance funds: extending universal health coverage in low- and middle-income countries of the WHO European Region
Source: Int J Equity Health. 2016 Apr 2;15:57. doi: 10.1186/s12939-016-0321-0 (PMC4818884; doi:10.1186/s12939-016-0321-0)
Supplement: Additional file 2: — Benefit package. Provides information about the health services covered by the benefit package for the exempted groups in each country [21, 23, 24, 26, 27, 30, 31, 34, 36, 38, 44, 46, 65, 67, 68, 70–73, 77, 98–103]. (PDF 69 kb) [file 12939_2016_321_MOESM2_ESM.pdf]

Additional File 2. Services in the benefit package

Country	Services covered for the exempted population groups	Scope of services for the exempt compared to contributors
Albania	only complementary to state-guaranteed benefit package (which offers specialized outpatient and inpatient care at public facilities free of charge for the entire population regardless of insurance status): basic services like family doctor's services, free visit to general practitioner, 90-100% of the cost of 12 tertiary unique examinations, primary and inpatient care in the city of Durres (pilot project since 2001)<br>dental: only emergency care, but broader package for patients < 18 years<br>pharmaceuticals: 410 drugs of the Essential Drug List fully/partially covered (44) (31)	more comprehensive: broader dental care package for patients < 18 years (44)
Bosnia & Herzegovina – *Federation*	comprehensive: primary care, specialized outpatient care, inpatient care<br>pharmaceuticals: drugs of the Essential Drug List covered (approx. 220 drugs, but number varies across cantons)<br>dental: n/a<br>other: refund of travel expenses incurred while seeking medical care (98) (34)	same (97) (34)
Bosnia & Herzegovina – *Republika Srpska*	information about the comprehensiveness of the benefit package n/a<br>pharmaceuticals: inpatient drugs, outpatient drugs of the Essential Drug List covered (220 drugs) (26)<br>dental: n/a	same (34)
Bulgaria	comprehensive: primary care, specialized outpatient care, inpatient care<br>dental: dental care partially covered<br>pharmaceuticals: inpatient and outpatient drugs on Positive Drug List and for certain disease fully/ partially covered (38)	same (38)
Georgia *(MIP)*	comprehensive: primary care, specialized outpatient care, inpatient care<br>pharmaceuticals: inpatient pharmaceuticals, outpatient pharmaceuticals partially covered up to 30 US$ annually (since 2010), drugs for some conditions (diabetes, Tb, HIV/AIDS) provided by the state without charges<br>dental care: not covered (21) (99)	Some differences in benefits packages and co-payments, but these are generally pro-poor (60)
Kyrgyzstan	only complementary to state- guaranteed benefit package (which offers emergency and primary care free of charge for the entire population)<br>the insured are exempt from co-payments to 60-66% of the average cost of treatment in hospitals, and to 50% of diagnostic costs in outpatient facilities; outpatient drugs from the Additional Drug Package covered (424 drugs in 2008) (30)	Same (30)
Lithuania	comprehensive: primary care, specialized outpatient care, inpatient care<br>dental: check-ups are free, but patients must pay for any work done<br>pharmaceuticals: drugs under the List of Compensated Medicines partially/ fully covered, full coverage of medication for chronic diseases that are prevalent among elderly people and medicines for grave diseases (cancer, TB, asthma, etc.) (77) (100)	same (32) (50)
Montenegro	comprehensive: primary care, specialized outpatient care, inpatient care<br>dental: only routine visits and preventive check-ups<br>pharmaceutical: drugs of the Positive Drug List covered<br>other: refund of travel expenses incurred while seeking medical care (67) (101) (102) (68)	same (67) (68)

Country	Services covered for the exempted population groups	Scope of services for the exempt compared to contributors
Republic of Moldova	complementary to state-guaranteed benefit package (which offers emergency and primary care free of charge regardless of insurance status, TB, HIV and mental health also provided free of charge): specialized outpatient care, inpatient care<br>dental: some services (mostly emergency care and preventive check-ups), additional dental care for children < 18 years and pregnant women<br>pharmaceuticals: limited range of outpatient pharmaceuticals fully/ partially covered (38 medicines for the treatment of common conditions) (26)	more comprehensive: additional dental care for children < 18 years and pregnant women (26)
Romania	comprehensive: primary care, specialized outpatient care, inpatient care<br>dental: preventive services and 88 additional procedures fully/ partially covered<br>pharmaceuticals: outpatient drugs on Positive Drug List fully/ partially covered (24)	same (24)
Russian Federation	comprehensive ("daily needs care"):<br>primary care, specialized outpatient care, inpatient care<br>dental: some services only for children, war veterans, and other special groups<br>pharmaceuticals: inpatient pharmaceuticals; outpatient pharmaceuticals only covered<br>for children < 3 years, children from large families < 6 years, disabled people, citizens affected by radiation in the Chernobyl disaster, certain medical conditions, retired individuals receiving minimum pensions, parents and wives of deceased military serviceman, war veterans (11% of population in 2008) (27)	more comprehensive: dental care and outpatient pharmaceuticals also covered for some groups within the exempt population (27)
Serbia	comprehensive: primary care, specialized outpatient care, inpatient care<br>dental: some services only covered only for children, people > 65 years, pregnant women, and in emergency cases<br>pharmaceuticals: inpatient drugs, outpatient prescription drugs covered (71) (70) (724)	more comprehensive: dental care for children, elderly > 65 years, and pregnant women (71)
TFYR Macedonia	comprehensive: primary care, specialized outpatient care, inpatient care<br>pharmaceuticals: inpatient and outpatient drugs in accordance to the List of Medicines covered<br>dental: some dental care<br>other: refund of travel expenses incurred while seeking medical care (46) (73)	more comprehensive: also medical rehabilitation of certain chronic non-communicable diseases for children < 18 years (73)
Turkey *(Green Card Scheme)*	comprehensive: primary care, specialized outpatient care, inpatient care<br>but only covered for services received in public facilities; exempted patients can only use private facilities in emergency cases or if public facilities are fully occupied<br>dental: inpatient and outpatient dental care (broad range of services)<br>pharmaceuticals: inpatient drugs, outpatient prescription drugs partially covered (103) (23)	same (23)
