# Supplementary material for: State budget transfers to health insurance funds: extending universal health coverage in low- and middle-income countries of the WHO European Region
Source: Int J Equity Health. 2016 Apr 2;15:57. doi: 10.1186/s12939-016-0321-0 (PMC4818884; doi:10.1186/s12939-016-0321-0)
Supplement: Additional file 3: — Cost-sharing mechanisms. Provides information regarding the cost-sharing mechanism and rates in each country [21, 23, 24, 26, 27, 30, 31, 34, 36, 38, 44, 65, 67, 68, 72, 77, 85, 104–106]. (PDF 71 kb) [file 12939_2016_321_MOESM3_ESM.pdf]

Additional File 3. Cost-sharing mechanisms

Country	Cost-sharing mechanism and rates for all members	Same/different compared to contributing insured population
Albania	Co-payments for outpatient services (insurance contributions in theory cover approx. 50% of service costs); co-insurance for pharmaceuticals (31)(104)	Different (less):<br>Infants < 1 year, invalids, and war veterans exempt from co-insurance for pharmaceuticals (44)
Bosnia & Herzegovina<br>*– Federation*	Co-payments for services (34)	Different (less):<br>Co-payments depend on the patient's social status and available resources (34) (105)
Bosnia & Herzegovina<br>*– Republika Srpska*	Cost-sharing for services (mechanism not specified) (34)	n/a
Bulgaria	User fees for services: 1% of the minimum monthly salary per outpatient visit and 2% of the minimum monthly salary per day of hospitalization (up to 10 bed-days per year),<br>Co-insurance for outpatient pharmaceuticals (38)	Different (less):<br>There is complete exemption from cost-sharing for children, pregnant women, individuals with income below a certain threshold, chronically sick patients, the unemployed, and some other groups (but no exemptions for laboratory tests and outpatient pharmaceuticals); Children deprived of parental care, prisoners, children < 18 years with mental disorders exempt from user fees for dental care (38)
Georgia<br>*(MIP)*	Benefit ceilings for defined services (e.g., 9,000 US$ for an operation); co-insurance for outpatient pharmaceuticals: 50% of the costs (21)	n/a
Kyrgyzstan	Co-insurance for services: 50% of the costs of all non-basic outpatient diagnostic tests (basic test defined as the most frequent 10 tests), 34-40% of the average costs of inpatient treatment (30)	Different (less):<br>Cost-sharing varies depending on exemption category, region, and disease profile (30)
Lithuania	Co-payments for dental care for adults,<br>Co-insurance for outpatient pharmaceuticals (66)	Different (less):<br>Various groups including disabled persons, people who are only partially able to work, people receiving social pensions are refunded 50% of the base price* of the refundable medicines;<br>Children < 18 years and persons recognized as unable to work exempt from co-insurance for outpatient pharmaceuticals (77)

Country	Cost-sharing mechanism and rates for all members	Same/different compared to contributing insured population
Montenegro	Co-payments for services: vary depending on the type of disease, diagnostic, treatment, and rehabilitation cost as well as health care level (67)	Different (less):<br>Children, pregnant women, women during delivery and one year after, social welfare beneficiaries as well as people with certain disease exempt from co-payments (68)(67)
Republic of Moldova	Co-insurance for pharmaceuticals (26)	Different (less):<br>Children < 18 years, pregnant women, diabetes patients exempt from co-insurance (26)
Romania	Co-payments for services: for long-stay care as well as some ambulatory services;<br>Co-insurance for some categories of pharmaceuticals: 10% or 50% of the reference price (24)	Different (less):<br>Pregnant women, postpartum mothers, and children exempt from co-insurance and co-payments (irrespective of their insurance status),<br>Children < 18 years and youths < 26 years if enrolled in any form of education exempt from co-payments for dental care (24)
Russian Federation	No cost-sharing, other than for outpatient pharmaceutical (27)	Same (27)<br>Specific population groups are exempted from fees for outpatient pharmaceuticals
Serbia	Co-insurance for services: up to 35% of the prices of health care services,<br>Co-insurance for pharmaceuticals (72)	Different (less):<br>Pregnant women, disabled people, the unemployed, material assistance recipients, elderly > 65 years are exempt from cost-sharing (85)
TFYR Macedonia	Co-payments for services (except emergency and primary care),<br>Co-insurance for pharmaceuticals; The maximum payment amount for total cost-sharing is 70% from the average net salary of the previous year (106)	Different (less):<br>The maximum payment amounts for total cost-sharing are 20%-40% from the average net salary of the previous year for the persons with lower incomes, children, and the elderly > 65 years,<br>Recipients of social assistance; persons placed in an institution for social protection or in another family, except for medicines prescribed at the PHC level and for the treatment abroad; persons with mental diseases in the psychiatry hospital and the mentally retarded persons without parent care; children with special needs; infants < 1 year; war-disabled persons and their families exempt from cost-sharing (106)
Turkey<br>*(Green Card Scheme)*	Co-payments for services: 4.8 US$ for outpatient visits with a discount of 1.8 US$ if no medicine is prescribed,<br>Co-insurance for outpatient pharmaceuticals: 20% of the costs (for retirees: 10%) (23)	Different (more):<br>Also co-payments for dental care, prosthetics, and orthotics (23)

** The base price for compensated medicines is set every year by the Ministry of Health and might differ from the actual retail price. The base price is reimbursed to a certain extent, whereas the difference between the base price and the retail price needs to be paid OOP.*
