# Supplementary material for: State budget transfers to health insurance funds: extending universal health coverage in low- and middle-income countries of the WHO European Region
Source: Int J Equity Health. 2016 Apr 2;15:57. doi: 10.1186/s12939-016-0321-0 (PMC4818884; doi:10.1186/s12939-016-0321-0)
Supplement: Additional file 4: — Incidence of catastrophic and impoverishing expenditure (at 40 % threshold level). Provides data regarding the incidence of catastrophic and impoverishing health expenditure [18, 49, 53, 55, 74, 90, 107]. (PDF 60 kb) [file 12939_2016_321_MOESM4_ESM.pdf]

Additional File 4. Incidence of catastrophic and impoverishing expenditure (at 40% threshold level)

Country	Catastrophic expenditure				Incidence of impoverishing expenditure
	Total population	Poorest quintile	Richest quintile	Only the exempted	
Albania (54) *at 25% threshold level*	8.1 (2002)<br>6.0 (2005)<br>5.4 (2008)	12.2 (2002)<br>13.2 (2005)<br>10.0 (2008)	4.1 (2002)<br>1.7 (2005)<br>2.9 (2008)	n/a	Increased by 6.5 pp in 2002, by 4.3 pp in 2005, by 3.6 pp in 2008
Bosnia & Herzegovina (18)	n/a	n/a	n/a	n/a	3% (2004)
Bulgaria	n/a	n/a	n/a	n/a	n/a
Georgia (52)	n/a	17.7 (2007)<br>27.0 (2010)	10.3 (2007)<br>20.6 (2010)	22.4 (2010)	n/a
Kyrgyzstan (107)	0.6 (2009)	n/a	n/a	n/a	n/a
Lithuania (107)	1.4 (2009)	n/a	n/a	n/a	n/a
Montenegro (18)	n/a	n/a	n/a	n/a	0.4 (2004)
Republic of Moldova (49)	n/a	4.1 (2007)<br>6.0 (2008)<br>3.1 (2010)<br>3.1 (2011)	3.2 (2007)<br>4.3 (2008)<br>6.4 (2010)<br>5.1 (2011)	n/a	n/a
Romania (107)	0.1 (2009)	n/a	n/a	n/a	n/a
Russian Federation (107)	6.0 (2009)	n/a	n/a	n/a	n/a
Serbia (107) (55)	2.3 (2007)<br>2.4 (2009)	5.6 (2007)	0.5 (2007)	n/a	1.2% (2003)<br>5.9% (2007)
TFYR Macedonia	n/a	n/a	n/a	n/a	n/a
Turkey (90)(74)	below 1%, but share of households increased from 2004-2010	Increased risk	Lower risk	1.7 (2003)<br>0.9 (2006)<br>0.9 (2009)	1.2% (2003)<br>2.2% (2006)<br>1.0% (2009)
